# Patterns and Potential Drivers of Dramatic Changes in Tibetan Lakes, 1972–2010

**DOI:** 10.1371/journal.pone.0111890

**Published:** 2014-11-05

**Authors:** Yingkui Li, Jingjuan Liao, Huadong Guo, Zewen Liu, Guozhuang Shen

**Affiliations:** 1 Department of Geography, University of Tennessee, Knoxville, Tennessee, United States of America; 2 Key Laboratory of Digital Earth Science, Institute of Remote Sensing and Digital Earth, Chinese Academy of Sciences, Beijing, P.R. China; Newcastle University, United Kingdom

## Abstract

Most glaciers in the Himalayas and the Tibetan Plateau are retreating, and glacier melt has been emphasized as the dominant driver for recent lake expansions on the Tibetan Plateau. By investigating detailed changes in lake extents and levels across the Tibetan Plateau from Landsat/ICESat data, we found a pattern of dramatic lake changes from 1970 to 2010 (especially after 2000) with a southwest-northeast transition from shrinking, to stable, to rapidly expanding. This pattern is in distinct contrast to the spatial characteristics of glacier retreat, suggesting limited influence of glacier melt on lake dynamics. The plateau-wide pattern of lake change is related to precipitation variation and consistent with the pattern of permafrost degradation induced by rising temperature. More than 79% of lakes we observed on the central-northern plateau (with continuous permafrost) are rapidly expanding, even without glacial contributions, while lakes fed by retreating glaciers in southern regions (with isolated permafrost) are relatively stable or shrinking. Our study shows the limited role of glacier melt and highlights the potentially important contribution of permafrost degradation in predicting future water availability in this region, where understanding these processes is of critical importance to drinking water, agriculture, and hydropower supply of densely populated areas in South and East Asia.

## Introduction

Rising temperature as a component of climate change has caused most glaciers worldwide to retreat [1], [2]. However, the significance of accelerated glacier melt to the water supply of high-latitude and high-altitude areas is still unclear [3]. As the highest and one of the most extensive plateaus on Earth, the Tibetan Plateau, along with its bordering mountains, has the largest number of glaciers outside of the Polar Regions, and these glaciers provide headwaters for most of Asia’s great river systems, including the Indus, Ganges, Brahmaputra, Mekong, Yellow, and Yangtze [4], [5]. Accelerated glacial retreat has the potential to affect the ecosystems and the livelihoods of more than 1.5 billion people in South and East Asia [4]–[7].

The Tibetan Plateau also contains >1,000 lakes, most of which belong to inland drainage systems [8], so their dynamics represent integrated effects of climate and associated cryospheric changes within their drainage basins. Recent lake expansions in many areas of the plateau have destroyed roads and infrastructure, flooded grazing and farm lands, and attracted significant attention from the scientific community [9]–[42]. Investigations of lake changes on the Tibetan Plateau during the past decades have been mainly based on remote sensing due to the lack of long-term systematic observation in this harsh environment [9]–[42]. Satellite images, such as Landsat MSS/TM/ETM+ imagery, have been used to investigate changes in lake extent [9]–[36]. Early studies mainly investigated one or a few lake(s) in the past several decades [9]–[33]. For example, Wang (2007) [14] investigated surface area variations of Nam Co (Tibet: “Co” = lake) and Selin Co from the central Tibetan Plateau, and several lakes in the source area of the Yellow River (northeastern Tibetan Plateau). They found that most lakes on the central plateau were expanding, whereas lakes in the source area of the Yellow River were shrinking. Wu and Zhu (2008) [19] conducted a similar study for Nam Co and indicated a continuous expansion of this lake in the past 40 years. They attributed the lake expansion to glacier retreat, the increase in temperature and precipitation, and the decrease in evaporation in the lake basin. Liu et al. (2009, 2010) [27], [28] detected changing patterns of three inland lakes on the central Tibetan Plateau (Nam Co, Zigetang Co, and Co Na) from 1965 to 2005 and correlated lake changes with changes in precipitation, glacial melting, and permafrost degradation. In recent years, more studies have started to investigate lake extent changes in relatively large areas and over the whole plateau [34]–[36]. For example, Huang et al. (2011) [34] investigated lake extent changes in the source areas of the Yellow and Yangtze Rivers on the northeastern Tibetan Plateau based on three time slices of Landsat images in the 1970s, early 1990s, and around 2004. They founded a widespread declining trend in lake abundance and area in the whole headwater basin of the Yellow River and the southeastern headwaters of the Yangtze River, but lake expansions occurred in the western and northern headwaters of the Yangtze River. Li et al. (2011) [35] conducted a plateau-wide lake change study using Landsat images of four time slices (the 1970s, around 1990, 2000, and 2009) and found that lake expansions dominated on the northern plateau, but lakes shrank in southern regions. The coarse temporal resolution prevented the identification of rapid/slow phases of lake changes and potential spatio-temporal transitions in lake changes.

Variations in lake levels can be monitored by satellite altimetry data [37]–[42]. Both satellite radar and ICESat altimetry data have been used to investigate recent lake-level variations for several large lakes on the Tibetan Plateau, such as Qinghai Lake [37] and Nam Co [38]. The results indicated that ICESat altimetry data are more precise than satellite radar altimetry and can be used to validate the inter-annual trends of lake-level change. Zhang et al. (2011) [39] examined lake level variations of >100 lakes across the Tibetan Plateau from 2003 to 2009 using ICESat altimetry data. They concluded that most lakes on the plateau were expanding because of glacier retreat in surrounding mountains, whereas the shrinkage of some lakes is mainly caused by decreasing precipitation. Phan et al. (2011) [40] conducted a similar study using the same dataset to examine more lake-level variations across the plateau. Zhang et al. (2013) [41] used ICESat altimetry data from lakes to assess the contribution of lake changes to the increased mass over the Tibetan Plateau derived by the Gravity Recovery and Climate Experiment (GRACE) data. However, as satellite altimetry data are limited to only within the recent decade (for example, ICESat altimetry data are available just from 2003 to 2009), a long-term plateau-wide assessment of lake changes is still lacking.

Although recent changes in lake extents and levels have been investigated using remote sensing techniques, the driving factors for the lake changes still remain unclear. Many studies have argued for the importance of accelerated glacier melting on recent lake changes (especially lake expansion) [10]–[12], [14]–[26], [29], [31], [39], [43], [44]. However, only few studies have estimated the contribution of glacier melting to the lake water balance, whereas the estimates have large uncertainties because of the difficulty in determining the volume change of glacial mass [29], [31]. Changes in precipitation and evaporation have also been proposed as major drivers for lake changes on the Tibetan Plateau [19], [21], [26]–[28], [30], [33], [41]. Some studies suggest that increasing precipitation leads to increasing cloud cover and humidity, decreasing sunshine duration and evaporation, and all these changes favor lake expansions on the central plateau [19], [26]–[28], [30]. In contrast, decreasing precipitation and increasing evaporation may lead to lake shrinkages on the southern plateau [20], [21]. A few studies also suggest permafrost degradation as a potential factor for lake changes [27], [28], [35], [36], but no detailed studies have been conducted to investigate the role of permafrost degradation on lake changes.

In the work reported here, we document a detailed record of lake extent and level changes since the 1970s across the Tibetan Plateau using the combination of Landsat imagery and ICESat altimetry data. Our aim is to determine if lakes across the Tibetan Plateau behave in a similar fashion or if distinct spatial and temporal patterns can be identified. Then, we test if the pattern of lake changes could be explained by spatial variations in glacial retreat. Finally, we examine the influences of other climate and cryospheric factors on the pattern of lake changes, including changes in precipitation/evaporation and the pattern of permafrost degradation.

## Datasets and Methods

**Figure 1:**
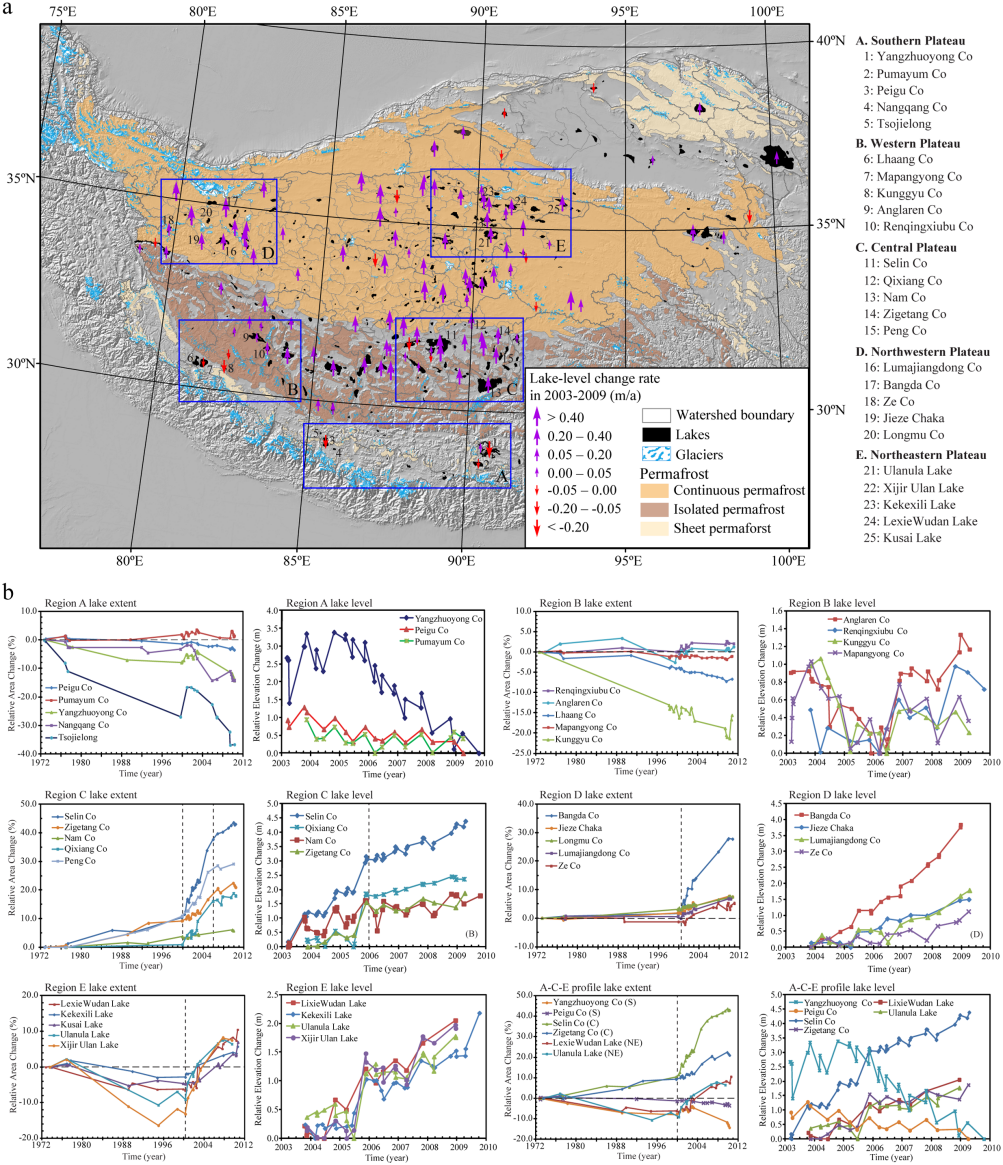
Lake changes across the Tibetan Plateau in recent decades. a, Spatial distribution of lake-level change rates (m/a) of 94 lakes (2003–2009) derived using ICESat altimetry. b, Detailed changes of lake extent since the 1970s (delineated using Landsat imagery) and lake levels in 2003–2009 for 25 lakes. These lakes were selected from five regions marked in panel a (5 lakes in each region: A-southern, B-western, C-central, D-northwestern, E-northeastern Tibetan Plateau). Changes in lake extents and levels along a profile from the southern (A), central (B), to northeastern (E) Tibetan Plateau are also illustrated in panel b.

We extracted detailed lake-level variations of 94 inland lakes that contain at least four years of measurements between 2003 and 2009 using ICESat altimetry data [45] (Table S1) across the Tibetan Plateau. To systematically examine lake changes beyond 2003–2009, we selected 25 lakes from five regions (five in each region, Fig. 1, Table S2) across the plateau to determine their detailed changes in extent from 1972 to 2010 using Landsat imagery (Tables S3–S7). Comparing the extents of these lakes over nearly four decades allows for an assessment of spatio-temporal patterns of lake change. We then compared these patterns with observed meteorological data from 1970 to 2010 within and around the plateau (Table S8). We also analyzed precipitation trends derived from the Tropical Rainfall Measurement Mission (TRMM) datasets [46] to supplement data from the relatively few meteorological stations on the northwestern plateau. We excluded lakes on ice surfaces and in front of modern glaciers because most of them drain downstream, and variable outflow from the system changes the relationship between changing lake levels and conditions within the drainage basin.

### Lake-level extraction based on ICESat altimetry data

We used ICESat altimetry data to extract lake levels in 2003–2009. ICESat was launched in January 2003 and used GLAS (the Geoscience Laser Altimeter System), a laser-ranging instrument, to measure land surface elevation in the world [45]. During its lifetime from 2003 to 2009, the GLAS instrument collected land surface elevations in designated periods each year (typically one in the spring (March) and one in the fall (September to October)). The available GLAS global land surface elevations have a vertical accuracy at the decimeter- to centimeter-level over flat surfaces and within an approximately 70 m diameter footprint [45], [47]. This dataset has the potential to monitor the changes in lake levels on the Tibetan Plateau. Because the distances between different ICESat tracks on the Tibetan Plateau are about 70 km [40], ICESat data are only available for the lakes that overlapped with ICESat tracks.

We developed a streamlined workflow to extract lake-level changes (Fig. S1a). We first imported GLAS data into ArcGIS as point features. Then, we extracted GLAS points within each lake using a lake polygon dataset provided by Chinese Academy of Sciences. Because the lake boundary may change over time, this operation may include some GLAS points outside the lakes, introducing outliers. In addition, instrument uncertainty or the impact of cloud or other factors may also introduce outliers. Based on the assumption that surface elevation is relatively constant for any given period across a lake, we designed a mode-mean method to extract lake elevations. As illustrated in Fig. S1b, the mode-mean method first examines the mode of all GLAS elevations based on a ±30 cm window (considering potential influences of waves on lake surface elevation) to exclude outliers and then determine the mean and standard deviation of the lake elevations. After extracting lake elevations for different periods, trend and regression analyses were conducted to determine the overall trends of the lake-elevation changes (the annual lake-level change rate (m/a)) and to identify detailed temporal patterns (Fig. S1c). All 94 inland lakes with at least 4 years of ICESat data are listed in Table S1, including the annual lake-level change rate (m/a), R^2^, and p-value. The uncertainty of the extracted lake surface elevation is <10 cm.

### Lake-extent delineation based on Landsat images

To systematically examine lake changes beyond the period of 2003–2009, we selected 25 lakes from five regions across the Tibetan Plateau to determine detailed changes in lake extent since the 1970s using Landsat MSS and TM/ETM+ images (Table S2). Landsat images were downloaded from the U.S. Geological Survey (USGS)’s Global Visualization Viewer (http://glovis.usgs.gov). To obtain a high temporal resolution of lake-extent changes, we examined and downloaded as many images as possible with a clear view of lake boundaries. In general, about 14–40 images were downloaded and processed for each lake (Tables S3–S7).

Most Landsat images were already geometrically and radiometrically corrected by the USGS EROS Digital Image Processing Center (http://seamless.usgs.gov/faq/nlcd_faq.php). For images that required additional geometric correction, we compared and geo-referenced them with other geometrically corrected images. Landsat MSS imagery has four spectral bands and the last two bands are sensitive to water bodies. TM/ETM+ images have seven and eight bands, respectively, and bands 4 and 5 are sensitive to water bodies [48]. Therefore, lake-boundary extraction was mainly based on these bands. Due to the distinct reflection difference between water and land, we extracted lake boundaries using thresholds automatically defined by the natural break classification in ArcGIS (Fig. S2a). To ensure the quality and accuracy, extracted lake boundaries were visually compared to the composite imagery (bands of 532 or 432 for the TM/ETM+ imagery) and erroneous boundaries were fixed by edit and additional manual digitization. Surface areas of each lake in different time slices were calculated in ArcGIS. Fig. S2b–c illustrates an example of lake boundaries extracted for Selin Co, the second largest lake on the Tibetan Plateau and the composite images that represent detailed lake extent changes in four different years of 1972, 1986, 1999, and 2010.

We assessed the uncertainty of Landsat image-delineated lake extent by comparing the lake boundary delineated using high resolution Google Earth imagery at a similar time slice. Different from the resolution of Landsat imagery (79 m for Landsat MSS and 30 m for TM/ETM+ imagery), the resolution of Google Earth imagery in some regions can reach to 0.6 m [49]. Since Google Earth images are usually a mosaic of multiple high resolution images taken at different times, we selected three relatively small lakes (Laxiong Co, Jieze Chaka, and Daze Co) completely located within the coverage of a single high resolution image to assess the uncertainty. We manually delineated the boundary of each lake from Google Earth (data source of the high resolution imagery: Digital Globe) and then compared it with a Landsat image-delineated lake boundary at a similar time slice (Fig. S3a–c). The comparison indicates that the uncertainty of Landsat image-delineated lake surface area is generally <1.0% and larger lakes tend to have smaller percentage area differences (Fig. S3d).

### Data sources and analysis of climate variables

**Figure 2:**
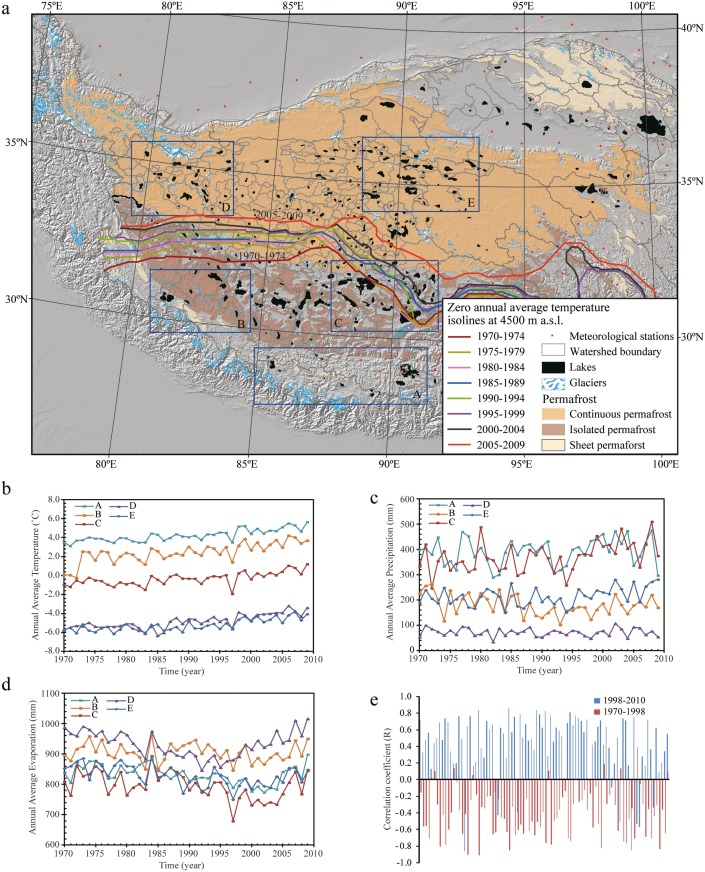
Climate variations on the Tibetan Plateau since the 1970s. a, Map showing the northward migration of 5-year annual average zero-temperature isolines (at 4500 m a.s.l). b, c, d, Variations in annual average temperature (b), precipitation (c), and evaporation (d) in five regions (A–E). e, Potential evapotranspiration trends before and after 1998 derived from meteorological stations within the Tibetan Plateau. The increasing or decreasing trends over time are represented by the correlation coefficient, R (R>0 indicates an increasing trend, and R<0 a decreasing trend).

We collected observed meteorological data (including daily mean temperature, precipitation, solar radiation, wind speed, and vapor pressure) for 1970–2010 from 117 stations within and around the Tibetan Plateau (Table S8) from the China Meteorological Data Sharing Service System (http://cdc.cma.gov.cn/). We calculated annual average temperature and annual precipitation for each station using the observed daily data. Since most stations have no directly observed evaporation data, we estimated the potential evapotranspiration of each station based on the Penman-Monteith model recommended by the Food and Agriculture Organization of the United Nations (FAO) [50]. We then applied the Kriging method to interpret the spatial distribution of annual temperature, precipitation, and potential evapotranspiration in each year across the plateau. A lapse rate of 6°C/1000 m was used to scale temperature to the surface of 4500 m above sea level (most lake levels are around this elevation) to interpret the spatio-temporal distribution of the temperature on this surface (Fig. S4). The spatial distributions of precipitation (Fig. S5) and potential evapotranspiration (Fig. S6) were interpreted without scaling due to the lack of lapse rates for these two variables. Since all climate variables are affected by local topography [51], the interpreted temperature, precipitation, and potential evapotranspiration may not represent the “real” values in each location. Instead, we mainly used the interpretations to examine the temporal trends of these variables across the Tibetan Plateau (Figs. 2, S7, S8).

Given the importance of precipitation in driving lake changes and the fact that few meteorological stations are located on the northwestern Tibetan Plateau, we further analyzed the Tropical Rainfall Measuring Mission (TRMM) Multi-satellite Precipitation Analysis 3B43 (Monthly precipitation data from 1998 to 2012, 0.25×0.25 degree merged TRMM, and other sources estimates) [46] to supplement the assessment of the role of precipitation on lake changes. This dataset combines the estimates generated by the TRMM and other satellites product (3B42) and the global gridded rain gauge data produced by NOAA’s Climate Prediction Center and/or the global rain gauge product produced by the Global Precipitation Climatology Center. Recent studies suggest that TRMM data, compared to rain gauge observations, show better performance than other satellite precipitation products with high correlation coefficients and lower root mean square errors [52]–[54]. We used the precipitation record from 92 stations within the plateau (with more than 10-years of precipitation record since 1998) to assess the quality of TRMM-derived precipitation and to examine the similarities and differences between the trends derived from these two data sources (Fig. S9). We also examined spatial patterns of TRMM-derived precipitation in 1998–2012 across the Tibetan Plateau (Fig. S10).

### Data sources and analysis of glacier and permafrost coverage for each lake basin

We delineated the drainage basin for each lake in ArcGIS using the 90-m resolution Shuttle Radar Topography Mission (SRTM) DEMs [55]. Then, we calculated the glacier and permafrost coverage (%) for each lake basin using the GLIMS glacier database [56] downloaded from the National Snow and Ice Data Center (http://nsidc.org/glims/) and the permafrost data [57] of the Tibetan Plateau downloaded from the Environmental and Ecological Science Data Center for West China (http://westdc.westgis.ac.cn/), respectively (Table S1).

## Results

### Patterns of lake change in 1972–2010

Our results in lake level and areal extent document both seasonal and inter-annual changes of Tibetan lakes (Figs. 1, S11). For example, seasonal changes of Selin Co in surface area can be identified using multiple Landsat TM images from different seasons (Fig. S11). Seasonal changes are also apparent in lake-level variations derived by ICESat altimetry data (Fig. S11). Lake level is usually low during winter or early spring and reaches its maximum in fall (September to October). Seasonal changes can also be observed in other lakes (Fig. 1b).

Beside seasonal changes, our results reveal a distinct southwest-northeast spatio-temporal pattern of lake inter-annual changes across the Tibetan Plateau in 1972–2010 from shrinking, to stable, to rapidly expanding (Fig. 1). Lakes on the southern and western plateau (Regions A and B in Fig. 1) have experienced continuous shrinkage or been relatively stable since the 1970s, except for a short period of slight expansion in 2000–2004. The lakes closest to the Himalayas have shrunk, whereas others have been relatively stable. On the central and northern plateau, most lakes expanded rapidly after 2000, but lake changes before 2000 varied with region. Lakes on the central plateau (Region C in Fig. 1) expanded continuously in 1972–2010, with expansion rates relatively limited (<10% in lake-extent change) before 2000, but rapid after 2000 (20–40%). A slowing trend in the expansion rate appeared after 2006. Lakes on the northwestern and northeastern plateau also expanded rapidly after 2000. However, before 2000, lakes on the northwestern plateau (Region D in Fig. 1) were stable or slightly shrinking and lakes on the northeastern plateau (Region E in Fig. 1) experienced various extents of shrinkage. In addition, no slowing trend occurred after 2006 in these two regions, and all lakes continue to expand rapidly. This relatively long-term record of lake changes indicates that lakes were primarily shrinking or relatively stable on the southern and western plateau, but expanding dramatically on the central and northern plateau after 2000, even after different patterns of lake change before 2000.

### Temperature variations across the Tibetan Plateau

The annual average temperature on the Tibetan Plateau has continuously increased since the 1970s, with a spatial pattern of much higher than 0°C distributed in the south, close to 0°C in the center, and below 0°C in the north. The temperature zone of close to 0°C (freezing point) has gradually migrated northward (Fig. 2a). The temperature trend, interpreted based on observed meteorological data, is consistent with the pattern of continuous increase in temperature after 2000 revealed using the land surface temperature (LST) product from Moderate Resolution Imaging Spectroradiometer (MODIS) [58].

### Precipitation variations across the Tibetan Plateau

Compared to the continuous increase in temperature, patterns of precipitation change are much more complex across the Tibetan Plateau. As illustrated in Figs. S5 and S10, precipitation on the Tibetan Plateau varies significantly among different years, but generally decreases from southeast to northwest. For the precipitation trend in each meteorological station (Fig. S7), during the period of 1970–2010, 75.3% of stations (64 out of 85 stations within the plateau that have >20 years of precipitation records) showed increasing trends, but only 15.3% of the trends (13 stations) were significant at the level of 0.05 (p<0.05). The other 21 stations (24.7%) showed decreasing trends, but none of them were significant. Because dramatic lake expansions on the central and northern plateau occurred mainly after 2000, we also analyzed the precipitation trends before and after 1998. For the period of 1970–1998, 58.1% of stations (50 out of 86 stations within the plateau with >15 years of precipitation records) showed increasing trends, but the increase was significant at only one station. The other 36 stations (41.9%) showed decreasing trends, but only one trend was significant. For the period of 1998–2010, 43.5% of stations (40 out of 92 stations within the plateau that have >10 years of precipitation records) showed increasing trends (but only 6 stations reached p<0.05). The other 52 stations (56.5%) showed decreasing trends (14 stations reached p<0.05). Our results are consistent with conclusions drawn by Yang et al. (2011) [59] that the precipitation trends on the Tibetan Plateau were not significant at p<0.05 although slightly increasing trends are observed in the semi-arid and semi-humid zones (central plateau) and decreasing trends in the humid and arid zones (southern and western plateau).

**Figure 3:**
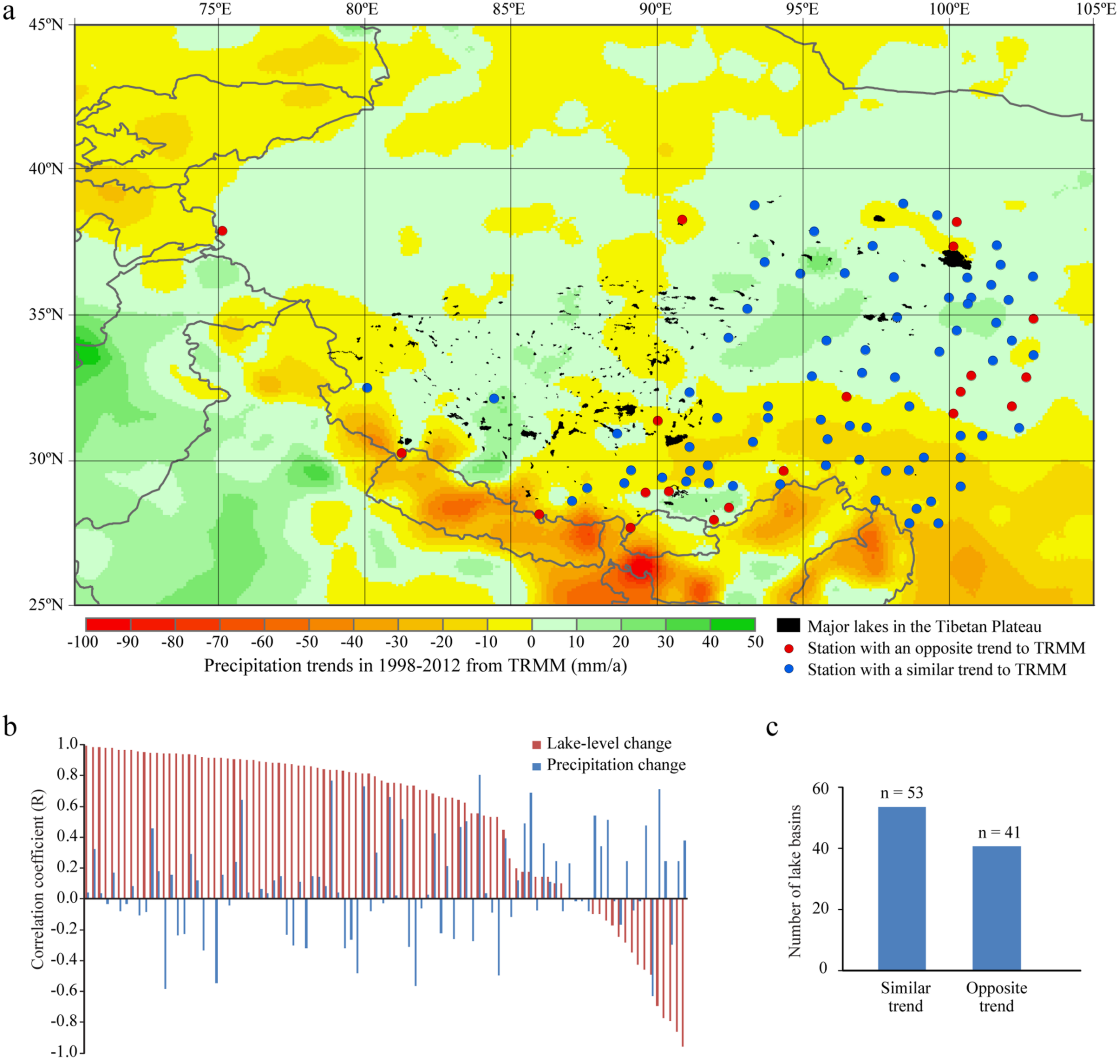
TRMM-derived precipitation trends in 1998–2012 on the Tibetan Plateau and its surrounding regions. a, Spatial distribution of TRMM-derived precipitation trends. b, Comparison between lake-level and precipitation trends for the 94 lake basins listed in Table S1. The increasing or decreasing trends over time are represented by the correlation coefficient, R. c, Numbers of lake basins with similar and opposite trends between lake-level and precipitation changes.

Our results indicate that TRMM-derived and station-observed precipitation are highly correlated (R^2^ = 0.79, Fig. S9a). Specifically, 90 out of 92 stations have positive correlations between these two datasets (p<0.05 for 76 stations, Fig. S9b). Such statistically significant correlations indicate that TRMM-derived data can be used to evaluate precipitation patterns on the Tibetan Plateau. Comparing precipitation trends derived for each station with its corresponding trend derived from the TRMM dataset also indicates that 72 out of 92 stations (78.3%) within the plateau have similar trends between these two datasets (Fig. S9c). The other 20 stations with opposite trends are located mainly in high-relief mountainous areas, such as several stations close to the Himalayas on the southern plateau and stations close to the eastern marginal mountains of the plateau (Fig. 3a). These results suggest that TRMM-derived data can be used to reflect precipitation trends in most areas of the Tibetan Plateau, especially relatively flat plateau surfaces on the central and northern plateau.

The TRMM-derived precipitation trends from 1998 to 2012 on the Tibetan Plateau and its surrounding regions (Fig. 3a) are similar to the trends derived using station-observed precipitation data. Specifically, precipitation on the southern and southwestern plateau is decreasing, especially close to the Himalayas. Precipitation on the central and northern plateau is slightly increasing in many regions, but decreasing in other regions, such as on the northwestern plateau, where five of our examined lakes are located (Fig. 1a), and in some areas on the central-northern plateau.

### Variations in potential evapotranspiration across the Tibetan Plateau

Chen et al. (2006) [60] analyzed the variations in potential evapotranspiration across the Tibetan Plateau using the Penman-Monteith method [50] and concluded that potential evapotranspiration in most areas of the plateau decreased from 1961 to 2000. They argued that the decreasing trend in potential evapotranspiration was mainly caused by the decreasing wind speed on the Tibetan Plateau [60]. We extended this analysis to 2010 using the same method and parameters (Figs. S6, S8a). Our results indicate that 72.6% of stations (61 out of 84 stations within the plateau that have >20 years of record) showed decreasing trends in potential evapotranspiration during the period of 1970–2010 (Fig. S8a) and 34.5% of the trends (29 stations) were significant (p<0.05). The other 23 stations (27.4%) showed increasing trends, but only the trends at 8 stations were significant (Fig. S8d). In 1970–1998 (Fig. S8b), 88.2% of stations (75 out of 85 stations within the plateau that have >15 years of record) showed decreasing trends, and the trends at 35 stations (41.2%) were significant. The other 10 stations (11.8%) showed increasing trends, but the increase was significant at only one station. These results are consistent with the conclusions drawn by Chen et al. (2006) [60].

However, in contrast to the decreasing trend before 1998, potential evapotranspiration showed increasing trends in most stations after 1998 (Fig. S8c). Specifically, 87.9% of stations (80 out of 91 stations within the plateau that have >10 years of record) showed increasing trends, with the trends at 42 stations (46.2%) being significant (p<0.05) (Fig. S8d). The other 11 stations (12.1%) showed decreasing trends (but only three of those were significant, Fig. S8d).

### Glacier and permafrost coverage over the Tibetan Plateau

Glaciers distributed on the Tibetan Plateau and surrounding mountains covered about 100,000 km^2^ in the 1970s [4]. A study of 7,090 glaciers suggested that the area of these glaciers decreased from ∼13,363 km^2^ in the 1970s to ∼12,130 km^2^ in the 2000s, with a total area reduction of 9.2% [4]. Glacial retreat is more apparent in the Himalayas and other marginal mountains, whereas the glaciers on the central plateau are relatively stable [4], [6], [61]. The glacier coverage of the 94 lake basins we investigated ranges from 0.0 to 10.5% and 29 lakes (mainly from the central and northern plateau) are free of glacier coverage in their drainage basins (Table S1).

The permafrost coverage is about 1.3×10^6 ^km^2^, accounting for ∼67% of the total area of the Tibetan Plateau [62], [63]. The thickness of the permafrost ranges from 10 m to 312 m [64] and the total volume of ground ice held in the permafrost is estimated at 9,528 km^3^, twice the volume of glaciers [65]. The permafrost coverage of the 94 lake drainage basins ranges from 7 to 100%. Fifty-five lake basins with 100% permafrost coverage are located on the central and northern Tibetan Plateau (Table S1).

## Discussion

Our derived changes in lake levels and extents are at least one order of magnitude higher than the uncertainties inherited from the Landsat/ICESat data. Therefore, the plateau-wide pattern of lake changes we derived represents the recent changes in water balances of these lakes driven by potential climate and associated cryospheric changes within their drainage basins.

**Figure 4:**
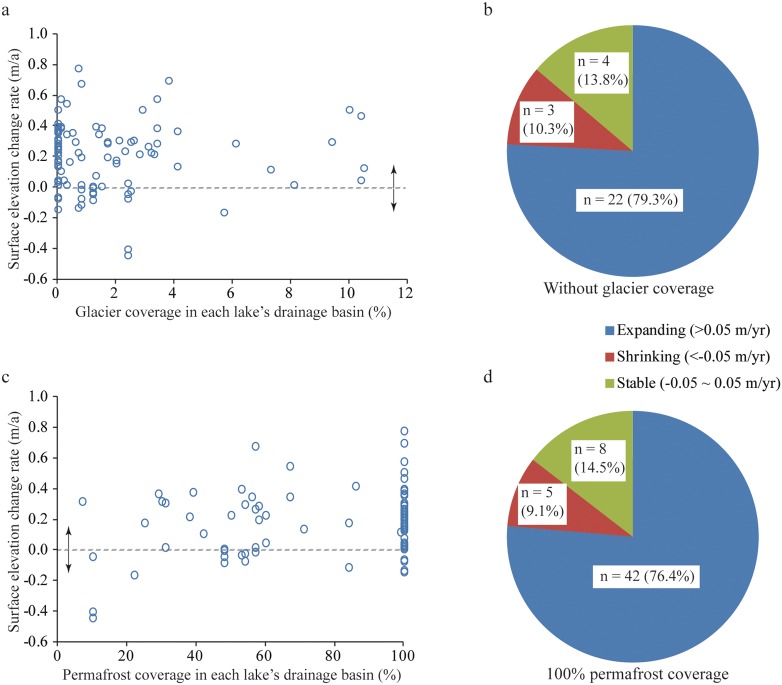
Relations between lake change and glacier/permafrost coverage in each lake’s drainage basin. a, Relations between lake-level change rate in 2003–2009 (m/a) and glacier coverage (%). b, Numbers of expanding, stable, and shrinking lakes in drainage basins with 0% glacier coverage. c, Relations between lake-level change rate in 2003–2009 (m/a) and permafrost coverage (%). d, Numbers of expanding, stable, and shrinking lakes in drainage basins with 100% permafrost coverage.

Temperature has continuously risen on the Tibetan Plateau since the 1970s, causing accelerated glacier melt (Fig. 2a–b). The rate of glacial retreat is more apparent in the Himalayas and other marginal mountains, with a decreasing trend from the margin to the center of the plateau [4], [6], [61]. However, our derived spatio-temporal pattern of lake changes across the Tibetan Plateau (Fig. 1) contrasts distinctly with the spatial characteristics of glacier retreat, suggesting limited influence of glacier melt on lake changes in this region. Specifically, our results indicate no statistically significant correlation between changes in lake levels (2003–2009) and glacier coverage in each lake’s drainage basin (Fig. 4a). Most lakes (>79%) in drainage basins containing no glaciers on the central and northern plateau still experienced expansion (Fig. 4b). In contrast, lakes on the southern plateau, fed by rapidly retreating glaciers, were relatively stable or shrinking. Therefore, our results suggest that glacier melt is not the dominant driver for the recent lake expansions across the Tibetan Plateau.

Meteorological records indicate a trend of stable or slightly decreasing precipitation on the southern, western, and northwestern plateau, but a slight increase in precipitation on the central and northeastern plateau (Fig. 2c). TRMM-derived precipitation trends in 1998–2012 show a similar pattern (Fig. 3a), but with more variability within the plateau. The overall pattern of lake changes across the plateau seems to be correlated with changing precipitation patterns, except for the northwestern plateau, where lakes expand rapidly after 2000 even under a slightly decreasing trend in precipitation. In particular, the relatively stable or slightly decreasing precipitation may explain the relative stability or slight shrinkage of lake extents/levels on the southern and western plateau, and the slight increase in precipitation seems to be related to the rapid expansions of most lakes on the central and northern plateau after 2000. Several recent studies [33], [41] have suggested the important role of precipitation in recent lake expansions. However, our detailed trend analysis indicates that not all lake-level trends in 2003–2009 of the 94 lakes we analyzed are directly related to the precipitation trends derived from the TRMM dataset. Specifically, only 56.4% of lakes we analyzed (53 out of 94) have similar (increasing or decreasing) trends between lake-level changes and precipitation variations in their corresponding drainage basins after 2000. In contrast, 43.6% of lakes (41 out of 94) have opposite trends (Fig. 3b–c). Although uncertainties exist in the TRMM dataset and TRMM-derived precipitation does not necessarily represent local precipitation observed from meteorological stations for all areas over the plateau, the comparison between these two datasets (Figs. 3a, S9) indicates that their derived precipitation trends are highly correlated (with positive correlations), especially on relatively flat plateau surfaces where our analyzed lakes and their basins are mainly located. Thus, our observation that 43.6% of lakes have opposite trends with precipitation is likely not caused by the TRMM dataset uncertainties. This indicates that the variations in precipitation alone may still not be able to fully explain the overall pattern of recent dramatic lake changes across the whole plateau.

The potential evapotranspiration estimated from meteorological stations decreased before 2000 [60], but showed a slightly increasing trend after 1998 in most stations (Figs. 2d–e, S8). The increasing evapotranspiration after 1998 would reduce surface flow into the lakes and mitigate the impact of potential precipitation increase on lake expansions. Therefore, recent dramatic expansions of most lakes on the central and northern plateau cannot be explained by changes in potential evapotranspiration.

The plateau-wide pattern of lake changes is consistent with the distribution of permafrost on the Tibetan Plateau (Fig. 1a). Due to its high elevation, 67% of the plateau is covered by permafrost that is sensitive to rising temperature [57], [62], leading to the increase in active layer thickness, thawing area, and the number of thawing days [66], [67]. Thus, permafrost degradation may play an important role in driving lake changes. When ground temperature is much lower than the melting point of the frozen soil, the water contribution from permafrost is limited and lake extents/levels are mainly controlled by other factors, such as the balance between precipitation and evaporation. When temperature increases to the melting point, accelerated permafrost melting will start to contribute to lake changes. However, if temperature continuously increases and remains above the melting point, the water contribution from permafrost will gradually become limited because water held in the frozen soil may already have been released. This supply-limited relationship between the amount of water contributed by permafrost and the ground temperature may explain the spatio-temporal pattern of lake changes across the Tibetan Plateau. The annual average temperature on the southern and western plateau has been much higher than 0°C since the 1970s (Fig. 2a–b); this may indicate that water contribution from permafrost was already limited and lake variations were mainly responding to changes in precipitation and evaporation. On the central plateau, temperature was lower than but close to the melting point before 2000; thus, lakes may have been able to expand slightly due to the contribution from the permafrost that was partially melted. Temperature in most areas of the central plateau might reach the melting point after 2000, increasing the permafrost contribution and causing rapid lake expansions. After 2006, continuously rising temperature may have reduced the permafrost contribution on the central plateau, slowing the trend of lake expansion. On the northern plateau, temperature was much lower than the melting point before 2000. Lake levels during this period remained relatively stable or shrank, depending on the water balance from other factors. Temperature on a significant portion of the northern plateau may have reached the melting point after 2000, causing accelerated permafrost melting and rapid lake expansion. Due to the relatively low temperature on the northern plateau, the permafrost contribution after 2006 may still be increasing, causing continuous lake expansion without a slowing trend.

This explanation is partially supported by the distribution of different permafrost types across the Tibetan Plateau (Fig. 1a). Permafrost in the southern plateau is discontinuous, sporadic and found mainly in higher mountains, whereas permafrost in the central and northern plateau is continuous and covers most plateau surfaces and mountains [57], [62]. Long-term permafrost monitoring on the Tibetan Plateau indicated that the permafrost area has been shrinking rapidly in response to rising temperature in recent decades [57], [62]. In particular, the lower elevation limit of permafrost moved up by 25 m in the north in the past 30 years and between 50 and 80 m in the south over the last 20 years [57], [62]. A previous study indicated that the active layer thickness of the permafrost along the Qinghai-Tibet Highway increased at a rate of 7.5 cm/a from 1995 to 2007 [66]. Furthermore, recent work showed that the active layer thickness increased at a rate of 3.1 cm/a from 1998 to 2010 for the permafrost distributed above 4700 m a.s.l [67]. Based on drilling data, a recent study found that ground ice is widely distributed in the Beiluhe Region, a permafrost monitoring area on the northern plateau [68]. This study also found that ice contents within 1–3 m beneath the permafrost table, where the increased active layer occurs, are >50% [68]. With the extensive drainage area feeding each lake (Table S1), permafrost thaw may contribute a significant amount of water to sustain the continuous lake expansion observed in the recent decade. Our results indicate a positive correlation between lake-level changes (2003–2009) and permafrost coverage in each lake’s drainage basin (Fig. 4c). In particular, >76% of lakes in drainage basins fully covered by permafrost had a clear trend of increasing lake levels (Fig. 4d). However, we recognized that not all permafrost melt would be converted into runoff flowing into lakes, and detailed studies of permafrost melt are of critical importance to quantify the water contribution from permafrost in the future.

Our observations from the Tibetan Plateau are consistent with the findings from the Arctic that lakes in continuous permafrost regions tend to expand, whereas lakes in isolated permafrost regions are shrinking [69], [70]. Our results also suggest that continuously rising temperature may eventually reduce the water contribution from permafrost, causing permafrost-driven lake expansions to cease. For example, the permafrost contribution to lake changes on the southern and western plateau may have already become limited due to high temperatures, and lake variations are mainly responding to the balance between precipitation and evaporation. The space-time transition of lake changes across the Tibetan Plateau suggests that the dramatic lake expansions on the central and northern plateau since 2000 are probably ephemeral and may not be sustained under continuously rising temperature. This finding has important implications for ecosystem, water resource, agriculture, and infrastructure management on the Tibetan Plateau and its surrounding regions.

## Supporting Information

Figure S1 **Lake-level extraction based on ICESat altimetry data.** a, Flowchart of lake surface elevation extraction method. b, The mode-mean method used to extract lake surface elevations. c, Lake surface elevation variations of Selin Co on the Central Tibetan Plateau from 2003 to 2009, representing a continuous increasing trend in surface elevation.
(TIF)

Figure S2 **Lake-extent delineation based on Landsat images.** a, Flowchart of lake-boundary extraction method. b, Lake-boundary variations of Selin Co representing a continuous lake expansion since the 1970s. c, Landsat images of three marked regions in panel b (A, B, C) around Selin Co, representing lake-boundary changes in four different years (1972, 1986, 1999, 2010).
(TIF)

Figure S3 **Accuracy assessment of Landsat image derived lake boundary for three lakes using Google Earth high resolution imagery.** a, Laxiong Co. b, Jieze Chaka. c, Daze Co. d, A comparison table representing the percentage difference in lake areas.
(TIF)

Figure S4 **Spatial variations of annual average temperature at 4500 m a.s.l. in different years since the 1970s across the Tibetan Plateau.** Black dots represent meteorological stations used for temperature interpretation. A lapse rate of 6°C/1000 m was used to scale all observed data to the surface of 4500 m a.s.l. and then the Kriging method was used to interpret the spatial distribution of the temperature in each year at this surface.
(TIF)

Figure S5 **Spatial variations of annual precipitation in different years since the 1970s across the Tibetan Plateau.** Black dots represent meteorological stations used for precipitation interpretation. The interpretation was simply based on the observed data from each station.
(TIF)

Figure S6 **Spatial variations of annual potential evapotranspiration in different years since the 1970s across the Tibetan Plateau.** Black dots represent meteorological stations used for interpretation.
(TIF)

Figure S7 **Precipitation trends observed in meteorological stations within the Tibetan Plateau.** a, Precipitation trends in 1970–2010. b, Precipitation trends in 1970–1998. c, Precipitation trends in 1998–2010. d, Percentage of stations with different precipitation trends and statistical significant levels.
(TIF)

Figure S8 **Potential evapotranspiration trends observed in meteorological stations on the Tibetan Plateau.** a, Potential evapotranspiration trends in 1970–2010. b, Potential evapotranspiration trends in 1970–1998. c, Potential evapotranspiration trends in 1998–2010. d, Percentage of stations with different potential evapotranspiration trends and statistical significant levels.
(TIF)

Figure S9 **Comparison between precipitation data derived from TRMM and observed from meteorological stations within the Tibetan Plateau.** a, Regression between these two datasets. b, Number of stations where the correlation between these two datasets with p<0.05 vs. p>0.05. c, Number of stations where precipitation are with similar vs. opposite trends between these two dataset.
(TIF)

Figure S10 **Spatial variations of TRMM-derived annual precipitation in different years from 1998 to 2012 across the Tibetan Plateau.** The spatial resolution of the TRMM dataset is 0.25×0.25 degree.
(TIF)

Figure S11 **Variations in surface area and elevation of Selin Co from 1999 to 2010 indicating both seasonal and inter-annual changes.**
(TIF)

Table S1 **Lake-level changes of 94 lakes from 2003 to 2009 derived using ICESat altimetry data and the glacier and permafrost coverage in each lake’s drainage basin.**
(DOCX)

Table S2 **Selected lakes to delineate lake-extent changes using Landsat images.**
(DOCX)

Table S3 **Lake-extent changes in the southern plateau (Region A) delineated using Landsat images.**
(DOCX)

Table S4 **Lake-extent changes in the western plateau (Region B) delineated using Landsat images.**
(DOCX)

Table S5 **Lake-extent changes in the central plateau (Region C) delineated using Landsat images.**
(DOCX)

Table S6 **Lake-extent changes in the northwestern plateau (Region D) delineated using Landsat images.**
(DOCX)

Table S7 **Lake-extent changes in the northeastern plateau (Region E) delineated using Landsat images.**
(DOCX)

Table S8 **Meteorological stations (117) within and around the Tibetan Plateau used in this study.**
(DOCX)
