# Supplementary figures and images for: Patterns and Potential Drivers of Dramatic Changes in Tibetan Lakes, 1972–2010
Source: PLoS One. 2014 Nov 5;9(11):e111890. doi: 10.1371/journal.pone.0111890 (PMC4221193; doi:10.1371/journal.pone.0111890)

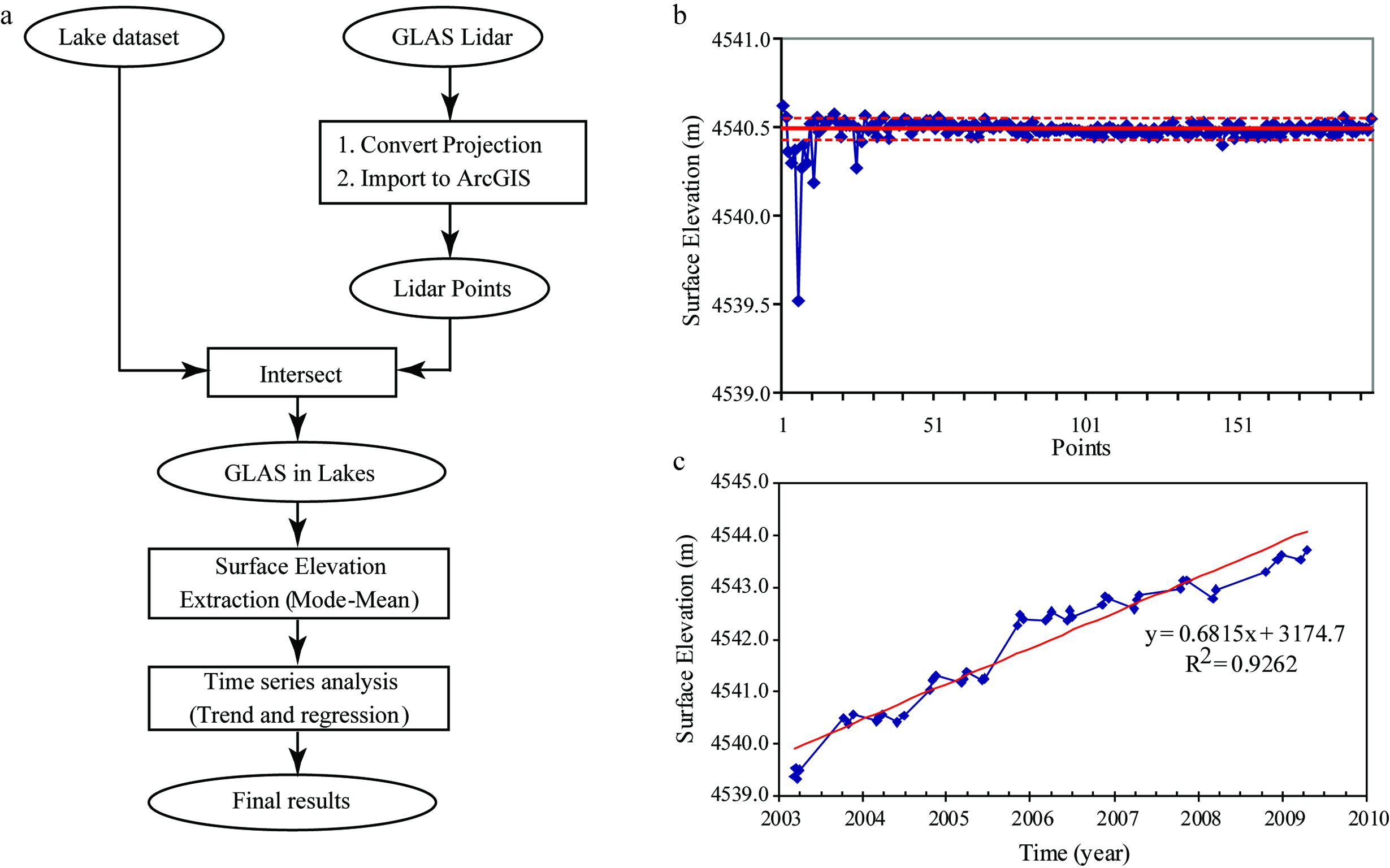

Supplement: Figure S1 — Lake-level extraction based on ICESat altimetry data. a, Flowchart of lake surface elevation extraction method. b, The mode-mean method used to extract lake surface elevations. c, Lake surface elevation variations of Selin Co on the Central Tibetan Plateau from 2003 to 2009, representing a continuous increasing trend in surface elevation. (TIF) [file pone.0111890.s001.tif]

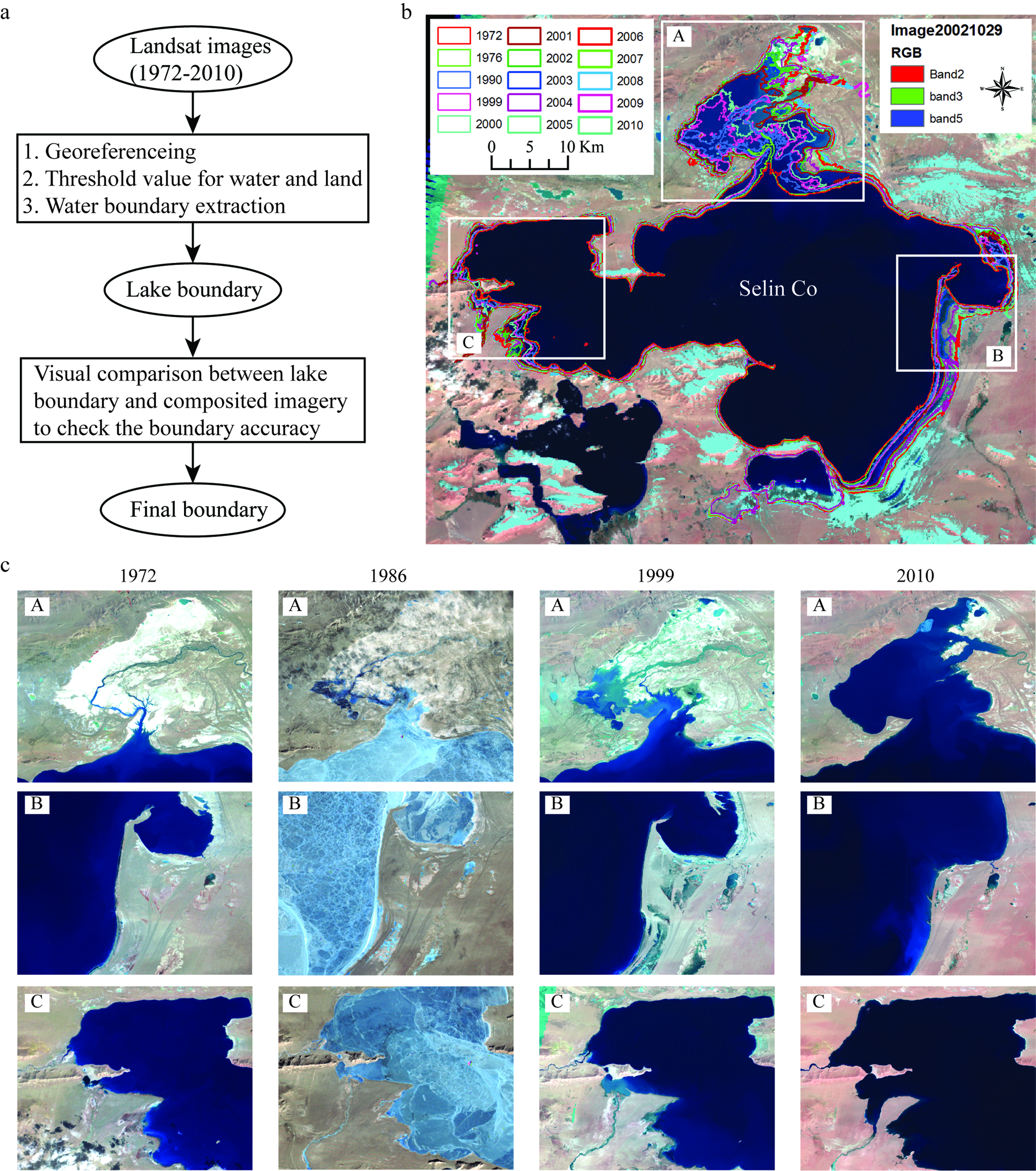

Supplement: Figure S2 — Lake-extent delineation based on Landsat images. a, Flowchart of lake-boundary extraction method. b, Lake-boundary variations of Selin Co representing a continuous lake expansion since the 1970s. c, Landsat images of three marked regions in panel b (A, B, C) around Selin Co, representing lake-boundary changes in four different years (1972, 1986, 1999, 2010). (TIF) [file pone.0111890.s002.tif]

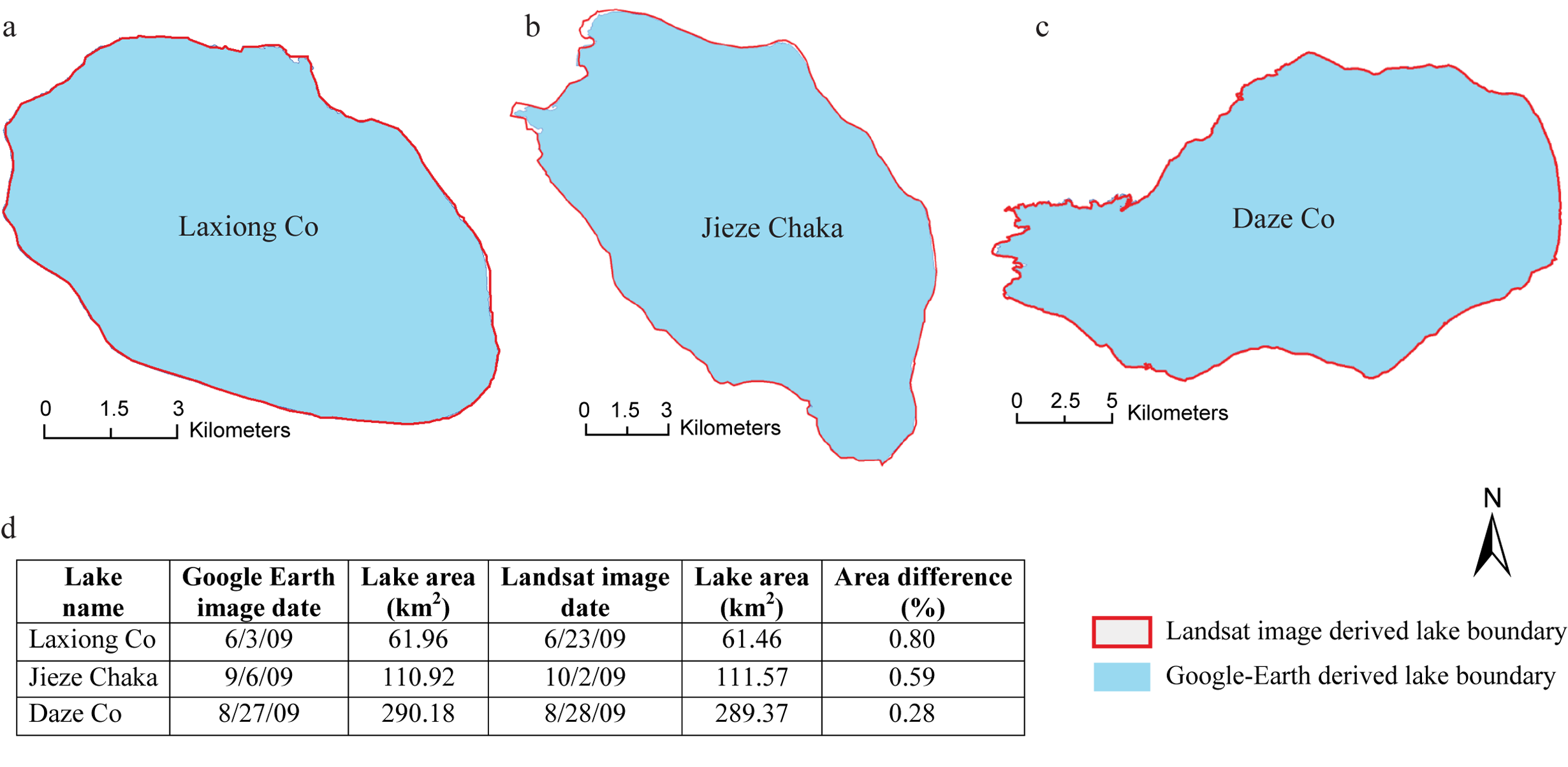

Supplement: Figure S3 — Accuracy assessment of Landsat image derived lake boundary for three lakes using Google Earth high resolution imagery. a, Laxiong Co. b, Jieze Chaka. c, Daze Co. d, A comparison table representing the percentage difference in lake areas. (TIF) [file pone.0111890.s003.tif]

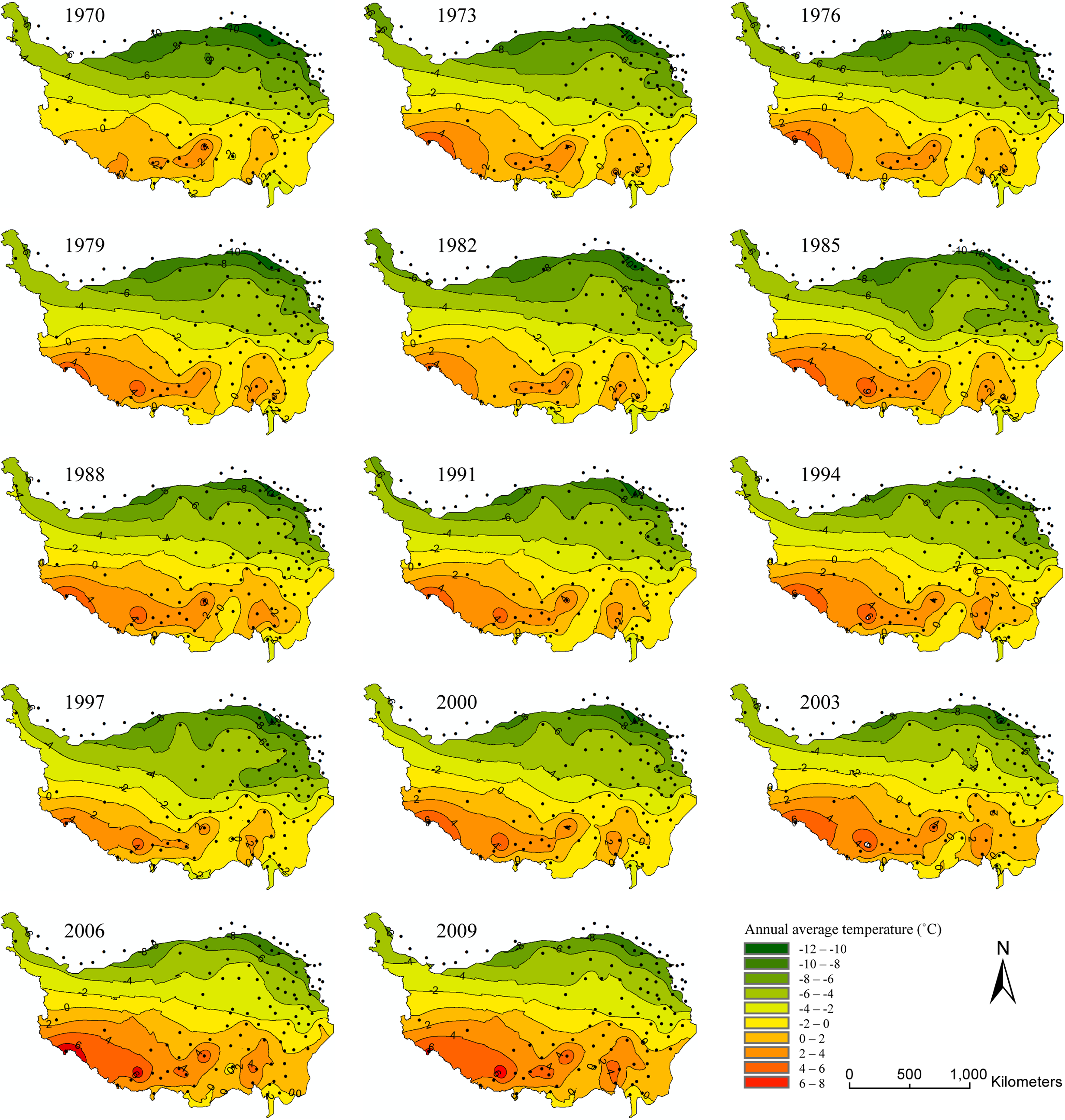

Supplement: Figure S4 — Spatial variations of annual average temperature at 4500 m a.s.l. in different years since the 1970s across the Tibetan Plateau. Black dots represent meteorological stations used for temperature interpretation. A lapse rate of 6°C/1000 m was used to scale all observed data to the surface of 4500 m a.s.l. and then the Kriging method was used to interpret the spatial distribution of the temperature in each year at this surface. (TIF) [file pone.0111890.s004.tif]

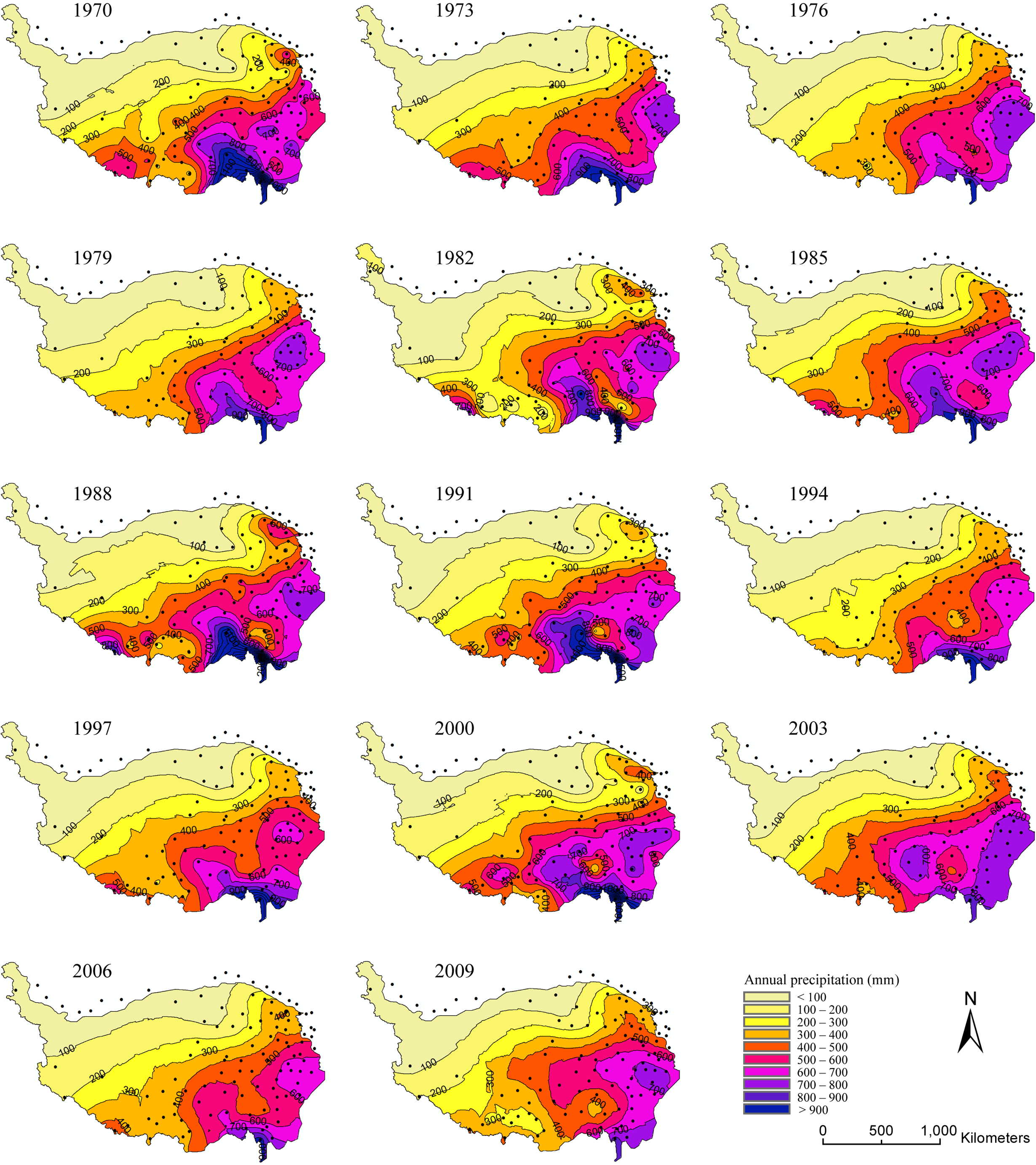

Supplement: Figure S5 — Spatial variations of annual precipitation in different years since the 1970s across the Tibetan Plateau. Black dots represent meteorological stations used for precipitation interpretation. The interpretation was simply based on the observed data from each station. (TIF) [file pone.0111890.s005.tif]

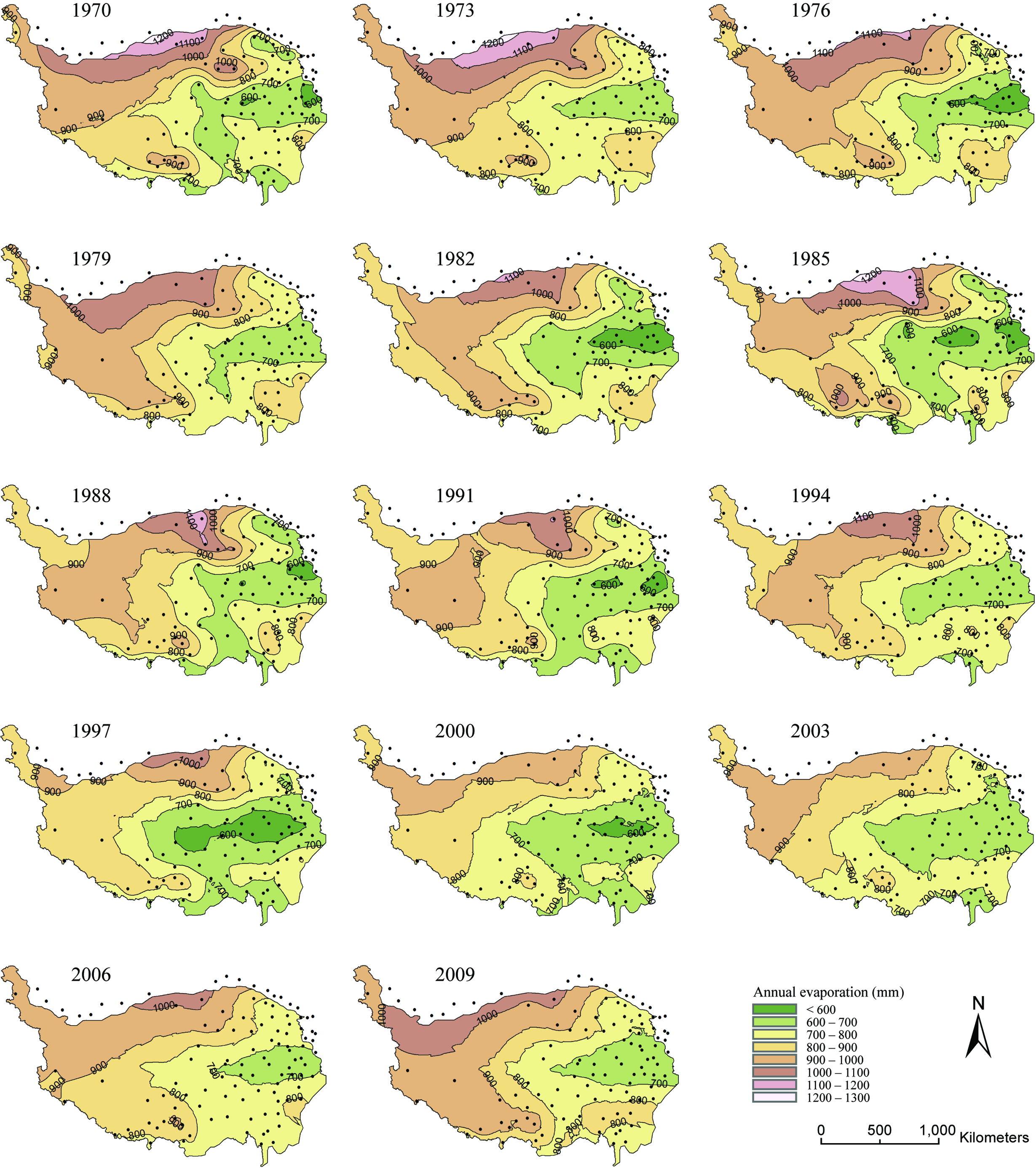

Supplement: Figure S6 — Spatial variations of annual potential evapotranspiration in different years since the 1970s across the Tibetan Plateau. Black dots represent meteorological stations used for interpretation. (TIF) [file pone.0111890.s006.tif]

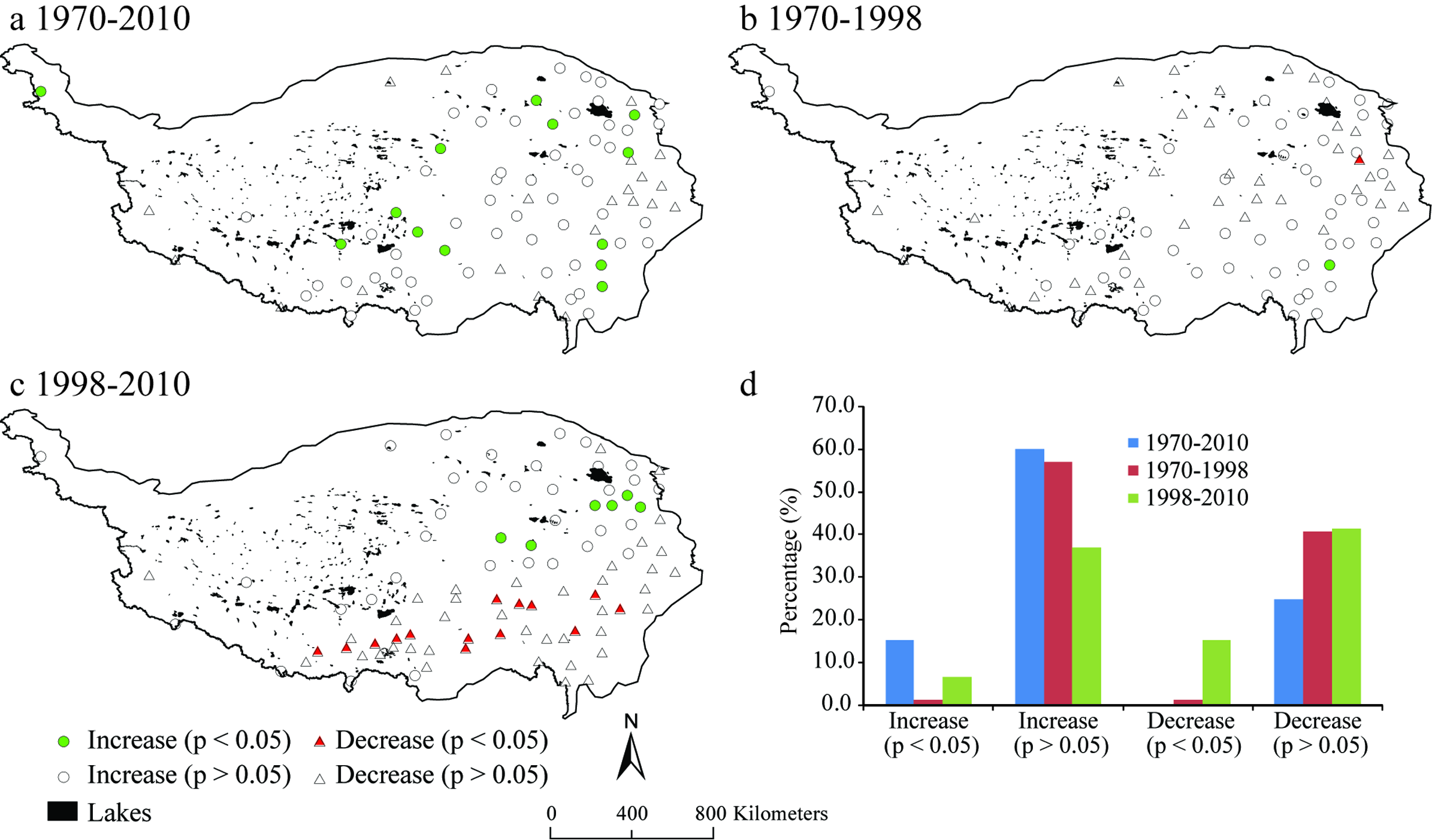

Supplement: Figure S7 — Precipitation trends observed in meteorological stations within the Tibetan Plateau. a, Precipitation trends in 1970–2010. b, Precipitation trends in 1970–1998. c, Precipitation trends in 1998–2010. d, Percentage of stations with different precipitation trends and statistical significant levels. (TIF) [file pone.0111890.s007.tif]

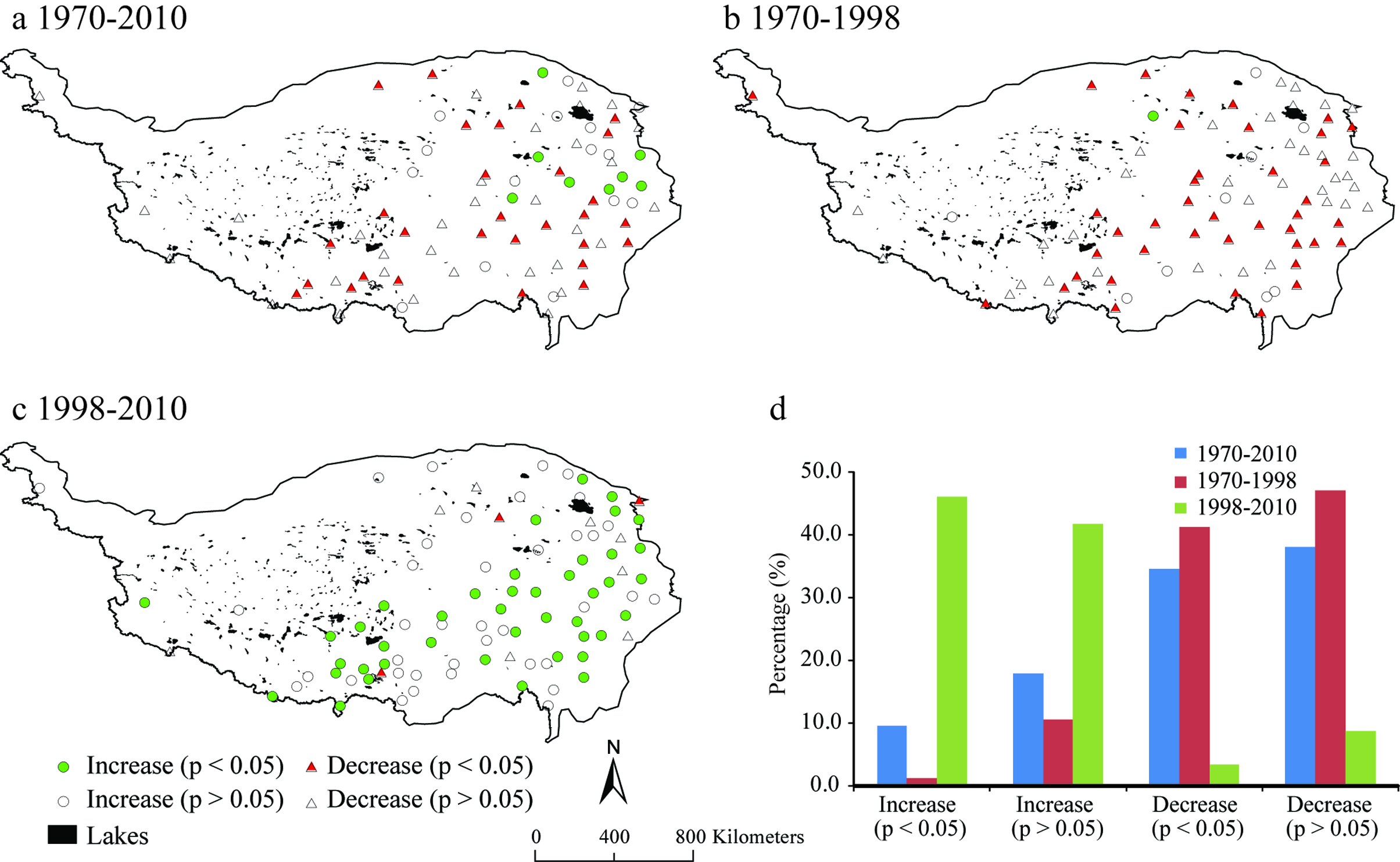

Supplement: Figure S8 — Potential evapotranspiration trends observed in meteorological stations on the Tibetan Plateau. a, Potential evapotranspiration trends in 1970–2010. b, Potential evapotranspiration trends in 1970–1998. c, Potential evapotranspiration trends in 1998–2010. d, Percentage of stations with different potential evapotranspiration trends and statistical significant levels. (TIF) [file pone.0111890.s008.tif]

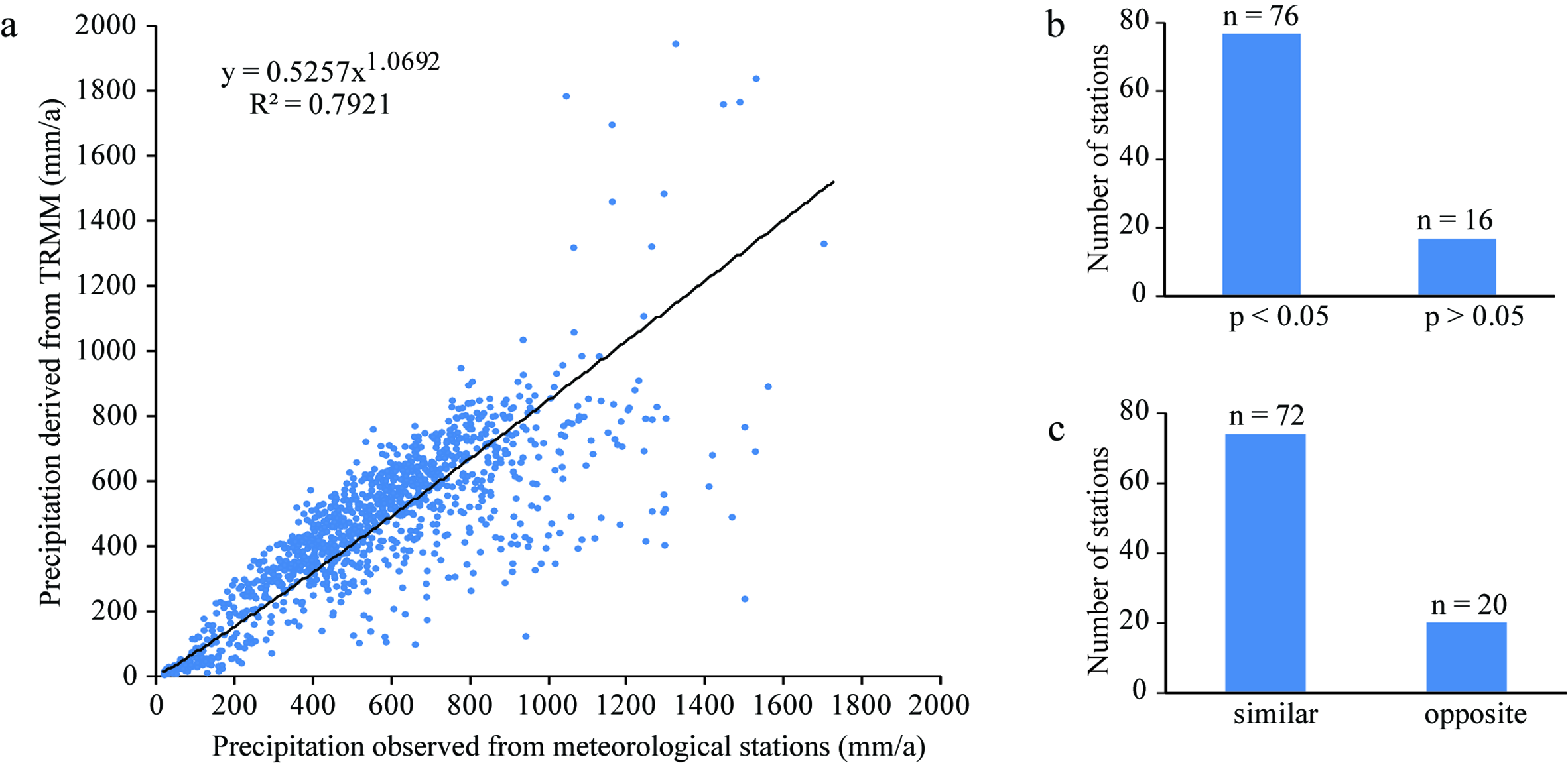

Supplement: Figure S9 — Comparison between precipitation data derived from TRMM and observed from meteorological stations within the Tibetan Plateau. a, Regression between these two datasets. b, Number of stations where the correlation between these two datasets with p<0.05 vs. p>0.05. c, Number of stations where precipitation are with similar vs. opposite trends between these two dataset. (TIF) [file pone.0111890.s009.tif]

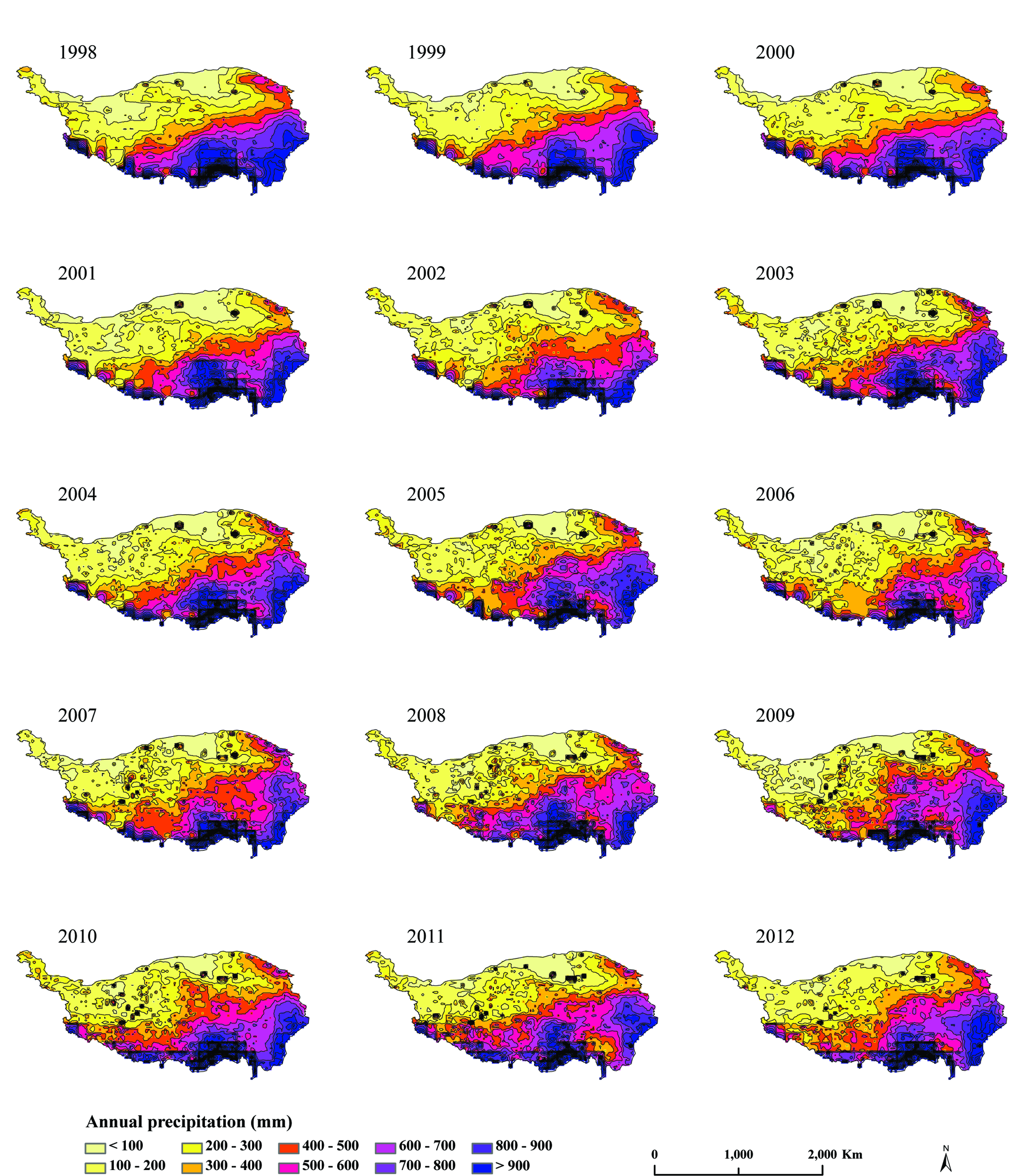

Supplement: Figure S10 — Spatial variations of TRMM-derived annual precipitation in different years from 1998 to 2012 across the Tibetan Plateau. The spatial resolution of the TRMM dataset is 0.25×0.25 degree. (TIF) [file pone.0111890.s010.tif]

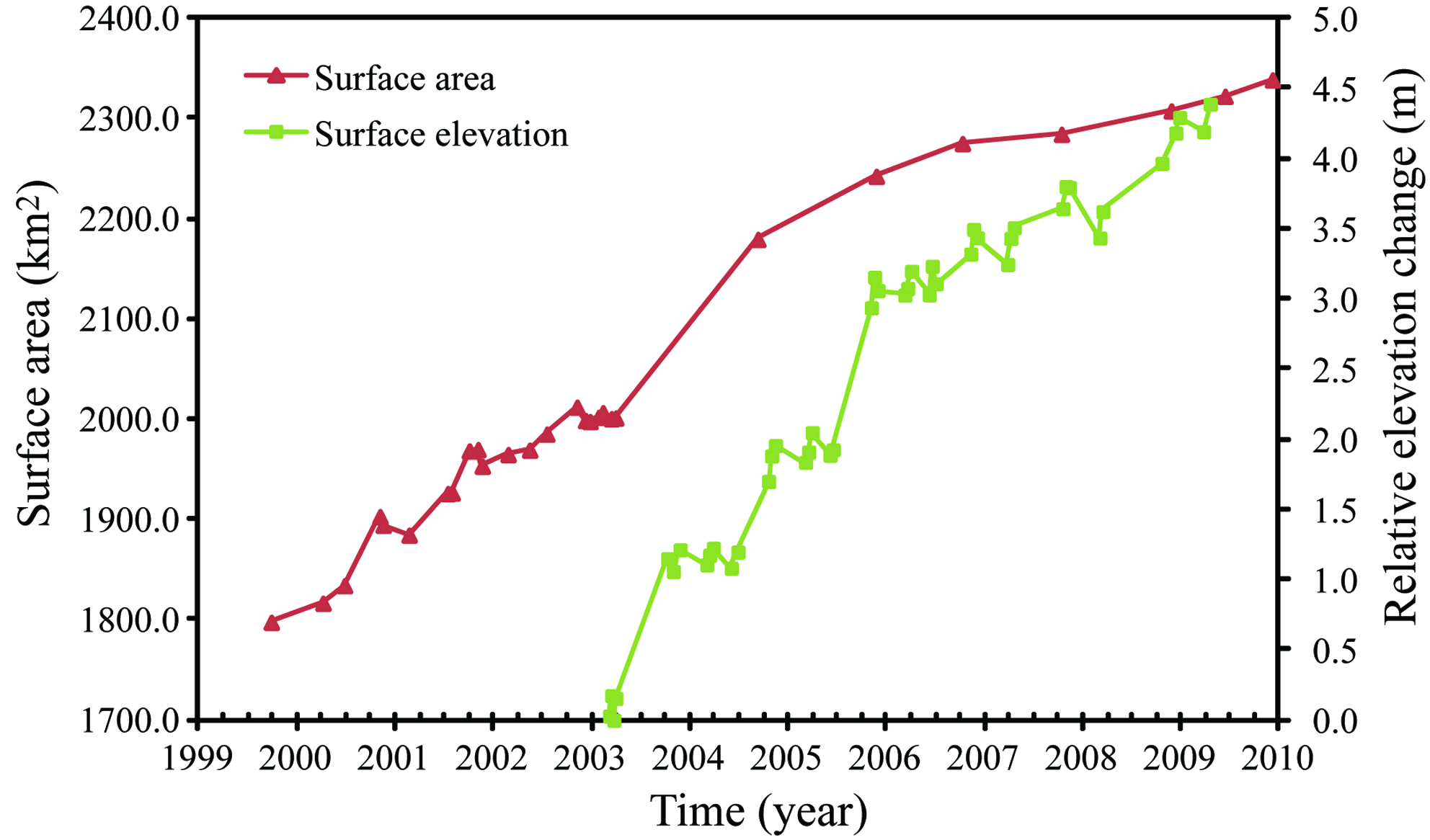

Supplement: Figure S11 — Variations in surface area and elevation of Selin Co from 1999 to 2010 indicating both seasonal and inter-annual changes. (TIF) [file pone.0111890.s011.tif]
