# Supplementary material for: Patterns and Potential Drivers of Dramatic Changes in Tibetan Lakes, 1972–2010
Source: PLoS One. 2014 Nov 5;9(11):e111890. doi: 10.1371/journal.pone.0111890 (PMC4221193; doi:10.1371/journal.pone.0111890)
Supplement: Table S1 — Lake-level changes of 94 lakes from 2003 to 2009 derived using ICESat altimetry data and the glacier and permafrost coverage in each lake’s drainage basin. (DOCX) [file pone.0111890.s012.docx]

**Table S1** Lake-level changes of 94 lakes from 2003 to 2009 derived using ICESat altimetry data and the glacier and permafrost coverage in each lake’s drainage basin

| ID | Lake  Name | Latitude  (˚) | Longitude  (˚) | lake-level  change rate  (m/a) | R^2^ | p-value | Basin-Lake ratio | Glacier  coverage | Permafrost  coverage |
| --- | --- | --- | --- | --- | --- | --- | --- | --- | --- |
| 1 | Aru Co | 33.99 | 82.40 | 0.05 | 0.04 | 0.49 | 10.6 | 0.104 | 1.00 |
| 2 | Peigu Co | 28.90 | 85.60 | -0.16 | 0.63 | 0.00 | 8.8 | 0.057 | 0.22 |
| 3 | Qinghai Lake | 36.89 | 100.19 | 0.11 | 0.57 | 0.00 | 7.0 | 0.001 | 0.42 |
| 4 | Kuhai Lake | 35.30 | 99.18 | -0.14 | 0.92 | 0.04 | 19.4 | 0.000 | 1.00 |
| 5 | Waxunggabma Co | 34.78 | 98.29 | 0.18 | 0.20 | 0.44 | 22.3 | 0.000 | 0.25 |
| 6 | Suhai Lake | 38.87 | 93.88 | -0.03 | 0.01 | 0.54 | 182.3 | 0.012 | 0.53 |
| 7 | Har Lake | 38.30 | 97.59 | 0.16 | 0.67 | 0.00 | 8.2 | 0.020 | 1.00 |
| 8 | Gasin Kol Lake | 38.12 | 90.78 | -0.07 | 0.06 | 0.53 | 208.6 | 0.008 | 0.54 |
| 9 | Ayakkuh Lake | 37.56 | 89.39 | 0.20 | 0.88 | 0.00 | 37.9 | 0.017 | 1.00 |
| 10 | Aqqik Kol | 37.08 | 88.42 | 0.31 | 0.66 | 0.00 | 38.5 | 0.021 | 1.00 |
| 11 | Keqikkum Kol | 36.98 | 90.74 | -0.06 | 0.01 | 0.86 | 44.0 | 0.000 | 1.00 |
| 12 | Yinbo Lake | 36.19 | 88.15 | 0.17 | 0.31 | 0.09 | 49.1 | 0.004 | 1.00 |
| 13 | Changhong Lake | 36.05 | 86.02 | 0.58 | 0.75 | 0.03 | 165.6 | 0.001 | 1.00 |
| 14 | Meiju Co | 36.03 | 88.51 | 0.39 | 0.76 | 0.00 | 44.5 | 0.001 | 1.00 |
| 15 | Xianhe Lake | 35.99 | 88.11 | 0.25 | 0.72 | 0.00 | 107.4 | 0.000 | 1.00 |
| 16 | Kushuihuan Lake | 35.99 | 90.12 | 0.17 | 0.50 | 0.01 | 10.5 | 0.000 | 1.00 |
| 17 | Xiangyang Lake | 35.81 | 89.42 | 0.36 | 0.90 | 0.00 | 15.3 | 0.005 | 1.00 |
| 18 | Yurbao Co | 35.74 | 86.70 | 0.51 | 0.90 | 0.00 | 49.9 | 0.029 | 1.00 |
| 19 | N. Heishi Lake | 35.57 | 82.76 | 0.30 | 0.97 | 0.00 | 21.8 | 0.094 | 1.00 |
| 20 | Deyu Co | 35.69 | 87.27 | -0.07 | 0.48 | 0.06 | 62.3 | 0.000 | 1.00 |
| 21 | LexieWudan Lake | 35.74 | 90.20 | 0.39 | 0.93 | 0.00 | 9.4 | 0.034 | 1.00 |
| 22 | Yueliang Lake | 35.61 | 90.41 | 0.26 | 0.79 | 0.02 | 36.0 | 0.000 | 1.00 |
| 23 | Kekexili Lake | 35.60 | 91.10 | 0.30 | 0.84 | 0.00 | 7.0 | 0.025 | 1.00 |
| 24 | Gaotai Lake | 35.42 | 90.97 | 0.08 | 0.70 | 0.04 | 17.8 | 0.013 | 1.00 |
| 25 | Chaoyang Lake | 35.28 | 87.26 | 0.04 | 0.03 | 0.58 | 108.1 | 0.000 | 1.00 |
| 26 | Xijir Ulan Lake | 35.21 | 90.34 | 0.40 | 0.81 | 0.00 | 17.8 | 0.013 | 1.00 |
| 27 | Zhailuorijiu Lake | 35.04 | 91.57 | 0.23 | 0.47 | 0.09 | 27.7 | 0.000 | 1.00 |
| 28 | Ulanula Lake | 34.81 | 90.48 | 0.30 | 0.80 | 0.00 | 13.2 | 0.006 | 1.00 |
| 29 | Luotuo Lake | 34.44 | 81.94 | 0.37 | 0.87 | 0.00 | 15.3 | 0.041 | 1.00 |
| 30 | Dogai Coring | 34.58 | 88.97 | 0.22 | 0.54 | 0.00 | 21.8 | 0.028 | 1.00 |
| 31 | Qagong Co | 34.43 | 82.34 | 0.40 | 0.99 | 0.00 | 29.7 | 0.000 | 1.00 |
| 32 | Goren Co | 34.60 | 92.46 | 0.05 | 0.03 | 0.55 | 39.3 | 0.000 | 1.00 |
| 33 | Co Nyi | 34.58 | 87.31 | 0.31 | 0.57 | 0.01 | 60.0 | 0.026 | 1.00 |
| 34 | Wenquan Lake | 34.43 | 83.56 | 0.15 | 0.31 | 0.08 | 120.3 | 0.000 | 1.00 |
| 35 | Memar Co | 34.22 | 82.31 | 0.47 | 0.78 | 0.00 | 10.6 | 0.104 | 1.00 |
| 36 | Marzharig Coqen | 34.34 | 91.59 | 0.05 | 0.39 | 0.18 | 31.3 | 0.000 | 1.00 |
| 37 | Pibi Lake | 34.20 | 87.79 | 0.02 | 0.01 | 0.76 | 80.6 | 0.000 | 1.00 |
| 38 | Gyado Co | 34.05 | 85.61 | 0.29 | 0.96 | 0.00 | 19.4 | 0.000 | 1.00 |
| 39 | Suana Lake | 33.92 | 86.69 | -0.13 | 0.21 | 0.26 | 50.5 | 0.007 | 1.00 |
| 40 | Kunchugchu Co | 33.72 | 82.67 | 0.24 | 0.28 | 0.22 | 22.8 | 0.023 | 1.00 |
| 41 | Longwei Co | 33.87 | 88.31 | 0.36 | 0.92 | 0.00 | 29.2 | 0.000 | 1.00 |
| 42 | Qoimo Co | 33.89 | 91.19 | 0.18 | 0.88 | 0.00 | 8.9 | 0.000 | 1.00 |
| 43 | Rumuchen Co | 33.74 | 90.64 | 0.27 | 0.75 | 0.00 | 14.1 | 0.031 | 1.00 |
| 44 | Meriqancomari | 33.64 | 89.71 | 0.39 | 0.84 | 0.00 | 26.0 | 0.015 | 1.00 |
| 45 | Chagbo Co | 33.37 | 84.19 | 0.21 | 0.78 | 0.00 | 96.5 | 0.000 | 1.00 |
| 46 | Duoersuodong Co east | 33.45 | 90.27 | 0.23 | 0.69 | 0.00 | 11.6 | 0.032 | 1.00 |
| 47 | Duoersuodong Co west | 33.40 | 89.86 | 0.58 | 0.89 | 0.00 | 9.8 | 0.034 | 1.00 |
| 48 | Qagam Co | 33.22 | 88.39 | 0.51 | 0.97 | 0.00 | 51.3 | 0.000 | 1.00 |
| 49 | Cedo Caka | 33.17 | 89.00 | 0.78 | 0.77 | 0.00 | 59.0 | 0.007 | 1.00 |
| 50 | Yaggain Canco | 33.02 | 89.79 | 0.21 | 0.43 | 0.01 | 29.5 | 0.000 | 1.00 |
| 51 | Darab Co | 32.47 | 83.21 | 0.23 | 0.41 | 0.01 | 104.7 | 0.000 | 0.60 |
| 52 | Qixiang Co | 32.50 | 90.00 | 0.42 | 0.89 | 0.00 | 18.5 | 0.000 | 0.86 |
| 53 | Ziru Co | 32.18 | 86.21 | 0.27 | 0.43 | 0.01 | 269.7 | 0.000 | 0.57 |
| 54 | Gyarab Co | 32.20 | 87.78 | 0.55 | 0.93 | 0.00 | 38.3 | 0.003 | 0.67 |
| 55 | Lagkor Co | 32.03 | 84.13 | 0.20 | 0.82 | 0.00 | 41.2 | 0.008 | 0.58 |
| 56 | Baqan Co | 31.93 | 82.78 | 0.14 | 0.68 | 0.04 | 36.3 | 0.000 | 1.00 |
| 57 | Zigetang Co | 32.10 | 90.90 | 0.32 | 0.74 | 0.00 | 17.4 | 0.000 | 0.30 |
| 58 | Gopug Co | 31.86 | 83.17 | 0.01 | 0.00 | 0.91 | 37.1 | 0.015 | 1.00 |
| 59 | Serbug Co | 32.00 | 88.23 | 0.35 | 0.96 | 0.00 | 38.3 | 0.003 | 0.67 |
| 60 | Cona | 32.03 | 91.48 | 0.05 | 0.07 | 0.67 | 25.1 | 0.002 | 0.60 |
| 61 | Xogor Co | 31.95 | 90.34 | 0.31 | 0.81 | 0.00 | 39.1 | 0.000 | 0.31 |
| 62 | Namka Co | 31.86 | 89.79 | 0.32 | 0.29 | 0.07 | 72.6 | 0.000 | 0.07 |
| 63 | Qagoi Co | 31.82 | 88.25 | -0.04 | 0.03 | 0.64 | 14.9 | 0.012 | 0.48 |
| 64 | Co Nag | 31.63 | 82.33 | 0.02 | 0.02 | 0.75 | 22.4 | 0.003 | 1.00 |
| 65 | Wuru Co | 31.72 | 88.00 | -0.08 | 0.18 | 0.08 | 14.9 | 0.012 | 0.48 |
| 66 | Anglaren Co | 31.60 | 83.00 | 0.03 | 0.02 | 0.46 | 22.9 | 0.024 | 1.00 |
| 67 | Selin Co | 31.80 | 89.00 | 0.68 | 0.93 | 0.00 | 20.3 | 0.008 | 0.57 |
| 68 | Jang Co | 31.55 | 90.82 | 0.38 | 0.82 | 0.00 | 10.1 | 0.000 | 0.39 |
| 69 | Co Ngoin | 31.59 | 88.72 | 0.02 | 0.01 | 0.72 | 13.5 | 0.008 | 0.57 |
| 70 | Renqingxiubu Co | 31.30 | 83.40 | 0.12 | 0.50 | 0.00 | 14.7 | 0.073 | 0.99 |
| 71 | Shib Co | 31.39 | 88.72 | -0.01 | 0.00 | 0.90 | 13.5 | 0.008 | 0.57 |
| 72 | Zigu Co | 31.38 | 87.90 | 0.01 | 0.00 | 0.93 | 14.9 | 0.012 | 0.48 |
| 73 | Dawa Co | 31.24 | 84.96 | 0.35 | 0.66 | 0.00 | 22.4 | 0.014 | 0.56 |
| 74 | Taruo Co | 31.14 | 84.12 | 0.30 | 0.70 | 0.00 | 25.6 | 0.017 | 0.54 |
| 75 | Argog Co | 30.99 | 82.24 | -0.07 | 0.12 | 0.26 | 22.9 | 0.024 | 1.00 |
| 76 | Geren Co | 31.12 | 88.34 | 0.00 | 0.00 | 0.98 | 14.9 | 0.012 | 0.48 |
| 77 | Angzi Co | 31.03 | 87.14 | 0.40 | 0.85 | 0.00 | 18.8 | 0.001 | 0.53 |
| 78 | Ringco Ogma | 30.93 | 89.67 | 0.37 | 0.59 | 0.01 | 17.4 | 0.000 | 0.29 |
| 79 | Kunggyu Co | 30.64 | 82.14 | -0.11 | 0.24 | 0.08 | 17.4 | 0.008 | 0.84 |
| 80 | Zhari Namco | 30.93 | 85.62 | 0.23 | 0.63 | 0.00 | 19.7 | 0.007 | 0.50 |
| 81 | Tangra Yumco | 31.07 | 86.61 | 0.29 | 0.44 | 0.00 | 11.2 | 0.017 | 0.58 |
| 82 | Nam Co | 30.70 | 90.60 | 0.22 | 0.54 | 0.00 | 5.5 | 0.033 | 0.38 |
| 83 | Dajia Co | 29.84 | 85.72 | 0.18 | 0.71 | 0.00 | 7.2 | 0.020 | 0.84 |
| 84 | Kongmu Co | 29.02 | 90.44 | 0.02 | 0.02 | 0.62 | 11.8 | 0.081 | 0.31 |
| 85 | Chen Co | 28.87 | 90.46 | -0.44 | 0.60 | 0.01 | 9.2 | 0.024 | 0.10 |
| 86 | Yangzhuoyong Co | 28.98 | 90.74 | -0.40 | 0.74 | 0.00 | 9.2 | 0.024 | 0.10 |
| 87 | Pumayum Co | 28.60 | 90.40 | -0.04 | 0.08 | 0.29 | 9.2 | 0.024 | 0.10 |
| 88 | Aksayqin Lake | 35.21 | 79.83 | 0.51 | 0.84 | 0.00 | 50.3 | 0.100 | 1.00 |
| 89 | Bangda Co | 34.96 | 81.56 | 0.70 | 0.91 | 0.00 | 18.7 | 0.038 | 1.00 |
| 90 | Ze Co | 34.20 | 79.80 | 0.13 | 0.28 | 0.04 | 16.5 | 0.105 | 1.00 |
| 91 | Jieze Chaka | 33.95 | 80.9 | 0.29 | 0.89 | 0.00 | 22.3 | 0.061 | 1.00 |
| 92 | Lumajiangdong Co | 34.04 | 81.63 | 0.29 | 0.83 | 0.00 | 25.3 | 0.034 | 1.00 |
| 93 | A'ong Co | 32.76 | 81.74 | 0.14 | 0.56 | 0.00 | 58.0 | 0.041 | 0.71 |
| 94 | Mapangyong Co | 30.68 | 81.46 | -0.02 | 0.02 | 0.56 | 11.0 | 0.025 | 0.54 |
