# Supplementary material for: Patterns and Potential Drivers of Dramatic Changes in Tibetan Lakes, 1972–2010
Source: PLoS One. 2014 Nov 5;9(11):e111890. doi: 10.1371/journal.pone.0111890 (PMC4221193; doi:10.1371/journal.pone.0111890)
Supplement: Table S2 — Selected lakes to delineate lake-extent changes using Landsat images. (DOCX) [file pone.0111890.s013.docx]

**Table S2** Selected lakes to delineate lake-extent changes using Landsat images

| ID | Lake Name | Latitude (˚) | Longitude (˚) | Area (km^2^)* | Elevation (m)** | Region*** | ICESat Data |
| --- | --- | --- | --- | --- | --- | --- | --- |
| 1 | Yangzhuoyong Co | 28.98 | 90.74 | 633.5 | 4447 | A | Y |
| 2 | Pumayum Co | 28.60 | 90.40 | 283.8 | 5019 | A | Y |
| 3 | Peigu Co | 28.90 | 85.60 | 277.6 | 4585 | A | Y |
| 4 | Nangqang Co | 28.72 | 85.89 | 256.9 | 4652 | A | N |
| 5 | Tsojielong | 29.12 | 85.40 | 175.8 | 4623 | A | N |
| 6 | Lhaang Co | 30.69 | 81.23 | 274.4 | 4575 | B | N |
| 7 | Mapangyong Co | 30.68 | 81.46 | 415.5 | 4590 | B | Y |
| 8 | Kunggyu Co | 30.63 | 82.14 | 64.7 | 4789 | B | Y |
| 9 | Anglaren Co | 31.60 | 83.00 | 507.3 | 4721 | B | Y |
| 10 | Renqingxiubu Co | 31.30 | 83.40 | 182.9 | 4765 | B | Y |
| 11 | Selin Co | 31.80 | 89.00 | 1628.8 | 4544 | C | Y |
| 12 | Qixiang Co | 32.50 | 90.00 | 153.0 | 4620 | C | Y |
| 13 | Nam Co | 30.70 | 90.60 | 1909.9 | 4729 | C | Y |
| 14 | Zigetang Co | 32.10 | 90.90 | 191.5 | 4573 | C | Y |
| 15 | Peng Co | 31.50 | 91.00 | 136.0 | 4534 | C | N |
| 16 | Lumajiangdong Co | 34.04 | 81.63 | 345.9 | 4817 | D | Y |
| 17 | Bangda Co | 34.96 | 81.56 | 102.9 | 4909 | D | Y |
| 18 | Ze Co | 34.20 | 79.80 | 112.8 | 4967 | D | Y |
| 19 | Jieze Chaka | 33.95 | 80.90 | 104.4 | 4530 | D | Y |
| 20 | Longmu Co | 34.60 | 80.40 | 96.4 | 5010 | D | N |
| 21 | Ulanula Lake | 34.81 | 90.48 | 544.1 | 4860 | E | Y |
| 22 | Xijir Ulan Lake | 35.21 | 90.34 | 373.7 | 4777 | E | Y |
| 23 | Kekexili Lake | 35.60 | 91.10 | 310.5 | 4891 | E | Y |
| 24 | LexieWudan Lake | 35.74 | 90.20 | 235.7 | 4875 | E | Y |
| 25 | Kusai Lake | 35.70 | 92.90 | 268.5 | 4480 | E | N |

*Lake area in the early 1970s; **Lake surface elevation from Google Earth; ***A-southern, B-western, C-central, D-northwestern, E-northeastern Tibetan Plateau
