# Supplementary material for: Patterns and Potential Drivers of Dramatic Changes in Tibetan Lakes, 1972–2010
Source: PLoS One. 2014 Nov 5;9(11):e111890. doi: 10.1371/journal.pone.0111890 (PMC4221193; doi:10.1371/journal.pone.0111890)
Supplement: Table S3 — Lake-extent changes in the southern plateau (Region A) delineated using Landsat images. (DOCX) [file pone.0111890.s014.docx]

**Table S3** Lake-extent changes in the southern plateau (Region A) delineated using Landsat images

| Yangzhuoyong Co | | Pumayum Co | | Peigu Co | | Nangqang Co | | Tsojielong | |
| --- | --- | --- | --- | --- | --- | --- | --- | --- | --- |
| Date | Area (km^2^) | Date | Area (km^2^) | Date | Area (km^2^) | Date | Area (km^2^) | Date | Area (km^2^) |
| 10/18/1972 | 633.5 | 10/18/1972 | 283.8 | 11/27/1972 | 277.6 | 09/16/1972 | 256.9 | 09/16/1972 | 175.8 |
| 04/17/1978 | 617.4 | 10/24/1976 | 286.6 | 12/15/1972 | 277.3 | 11/14/1976 | 255.4 | 10/28/1976 | 161.6 |
| 01/19/1989 | 588.5 | 11/11/1976 | 286.4 | 01/02/1973 | 277.6 | 05/13/1977 | 250.3 | 05/14/1977 | 156.5 |
| 10/30/1999 | 583.0 | 09/19/1989 | 283.2 | 10/28/1976 | 280.4 | 09/16/1977 | 250.1 | 08/16/1999 | 128.4 |
| 12/01/1999 | 584.9 | 10/30/1999 | 288.3 | 11/14/1976 | 279.6 | 11/30/1991 | 250.2 | 11/22/2000 | 146.6 |
| 04/07/2000 | 577.9 | 12/01/1999 | 287.9 | 12/02/1976 | 279.6 | 03/08/1993 | 245.3 | 09/22/2001 | 146.6 |
| 09/30/2000 | 600.9 | 02/19/2000 | 286.0 | 12/20/1976 | 278.9 | 08/16/1999 | 248.6 | 05/04/2002 | 145.2 |
| 11/17/2000 | 601.2 | 03/06/2000 | 285.6 | 01/07/1977 | 277.0 | 11/22/2000 | 252.2 | 11/28/2002 | 144.3 |
| 12/19/2000 | 600.3 | 04/07/2000 | 285.1 | 04/25/1977 | 276.4 | 09/22/2001 | 251.9 | 11/12/2005 | 136.1 |
| 02/05/2001 | 595.3 | 09/30/2000 | 290.6 | 05/13/1977 | 276.9 | 05/04/2002 | 247.6 | 10/14/2006 | 127.9 |
| 11/04/2001 | 599.8 | 11/17/2000 | 290.5 | 11/30/1991 | 276.3 | 11/28/2002 | 240.1 | 10/30/2006 | 128.4 |
| 01/23/2002 | 595.5 | 12/19/2000 | 290.3 | 12/06/1999 | 273.8 | 11/12/2005 | 220.4 | 05/15/2009 | 119.2 |
| 02/24/2002 | 591.8 | 02/05/2001 | 289.0 | 11/22/2000 | 274.6 | 10/30/2006 | 221.5 | 06/16/2009 | 111.1 |
| 10/22/2002 | 605.4 | 03/09/2001 | 287.9 | 09/22/2001 | 275.5 | 06/16/2009 | 228.7 | 04/16/2010 | 111.4 |
| 11/07/2002 | 608.6 | 11/04/2001 | 291.2 | 11/28/2002 | 274.3 | 08/19/2009 | 226.0 |  |  |
| 12/09/2002 | 608.5 | 01/23/2002 | 289.8 | 02/16/2003 | 273.3 | 10/22/2009 | 223.5 |  |  |
| 01/10/2003 | 606.7 | 07/18/2002 | 289.5 | 02/12/2005 | 271.4 | 11/23/2009 | 220.6 |  |  |
| 03/15/2003 | 603.9 | 10/22/2002 | 293.5 | 11/12/2005 | 271.4 | 03/31/2010 | 220.9 |  |  |
| 10/17/2009 | 559.4 | 12/09/2002 | 292.6 | 10/14/2006 | 272.3 | 04/16/2010 | 220.3 |  |  |
| 11/18/2009 | 556.6 | 01/10/2003 | 291.9 | 10/30/2006 | 272.2 |  |  |  |  |
| 01/21/2010 | 551.7 | 03/15/2003 | 290.7 | 05/15/2009 | 269.0 |  |  |  |  |
| 02/06/2010 | 548.8 | 05/05/2007 | 287.2 | 06/16/2009 | 268.5 |  |  |  |  |
| 04/11/2010 | 543.2 | 09/15/2009 | 291.6 | 10/22/2009 | 270.2 |  |  |  |  |
|  |  | 10/01/2009 | 292.1 | 11/07/2009 | 269.5 |  |  |  |  |
|  |  | 10/17/2009 | 292.2 | 11/23/2009 | 269.6 |  |  |  |  |
|  |  | 11/18/2009 | 290.8 | 12/09/2009 | 269.3 |  |  |  |  |
|  |  | 01/21/2010 | 288.7 | 03/31/2010 | 267.9 |  |  |  |  |
|  |  | 02/06/2010 | 288.1 |  |  |  |  |  |  |
|  |  | 02/22/2010 | 286.3 |  |  |  |  |  |  |
|  |  | 04/11/2010 | 287.2 |  |  |  |  |  |  |
