# Supplementary material for: Patterns and Potential Drivers of Dramatic Changes in Tibetan Lakes, 1972–2010
Source: PLoS One. 2014 Nov 5;9(11):e111890. doi: 10.1371/journal.pone.0111890 (PMC4221193; doi:10.1371/journal.pone.0111890)
Supplement: Table S4 — Lake-extent changes in the western plateau (Region B) delineated using Landsat images. (DOCX) [file pone.0111890.s015.docx]

**Table S4** Lake-extent changes in the western plateau (Region B) delineated using Landsat images

| Lhaang Co | | Mapangyong Co | | Kunggyu Co | | Anglaren Co | | Renqingxiubu Co | |
| --- | --- | --- | --- | --- | --- | --- | --- | --- | --- |
| Date | Area (km^2^) | Date | Area (km^2^) | Date | Area (km^2^) | Date | Area (km^2^) | Date | Area (km^2^) |
| 09/19/1972 | 274.4 | 09/19/1972 | 415.5 | 09/10/1972 | 64.7 | 11/24/1973 | 507.3 | 11/24/1973 | 182.9 |
| 10/31/1976 | 273.3 | 10/31/1976 | 416.5 | 05/06/1998 | 56.2 | 10/12/1976 | 505.9 | 11/17/1976 | 182.5 |
| 06/22/1977 | 270.0 | 12/06/1976 | 415.9 | 06/07/1998 | 55.6 | 11/17/1976 | 505.0 | 01/22/1989 | 183.9 |
| 10/23/1990 | 272.1 | 06/22/1977 | 414.6 | 10/13/1998 | 55.9 | 01/22/1989 | 510.0 | 11/02/1999 | 183.0 |
| 06/07/1998 | 263.6 | 10/23/1990 | 416.1 | 04/07/1999 | 55.2 | 07/13/1999 | 485.0 | 02/22/2000 | 182.7 |
| 10/13/1998 | 264.7 | 05/06/1998 | 410.6 | 09/22/1999 | 56.1 | 11/02/1999 | 489.8 | 06/13/2000 | 183.1 |
| 09/22/1999 | 263.2 | 06/07/1998 | 409.4 | 05/03/2000 | 54.2 | 05/12/2000 | 488.4 | 02/08/2001 | 183.9 |
| 05/03/2000 | 263.7 | 10/13/1998 | 411.5 | 09/24/2000 | 55.0 | 10/03/2000 | 495.8 | 07/02/2001 | 184.3 |
| 08/07/2000 | 262.8 | 09/22/1999 | 411.4 | 10/10/2000 | 55.9 | 07/02/2001 | 492.8 | 10/22/2001 | 185.1 |
| 09/24/2000 | 263.4 | 05/03/2000 | 410.1 | 08/26/2001 | 55.5 | 10/22/2001 | 493.0 | 03/15/2002 | 185.1 |
| 10/10/2000 | 263.2 | 07/06/2000 | 409.1 | 06/10/2002 | 54.8 | 03/15/2002 | 494.0 | 10/25/2002 | 186.8 |
| 05/06/2001 | 261.3 | 08/07/2000 | 411.2 | 09/30/2002 | 55.8 | 10/25/2002 | 499.1 | 06/30/2009 | 186.8 |
| 06/01/2002 | 260.5 | 10/10/2000 | 412.4 | 05/28/2003 | 53.7 | 06/30/2009 | 497.6 | 09/18/2009 | 187.7 |
| 09/30/2002 | 260.7 | 05/06/2001 | 410.9 | 06/05/2009 | 52.5 | 09/18/2009 | 499.4 | 10/20/2009 | 187.7 |
| 05/28/2003 | 260.8 | 08/10/2001 | 411.3 | 08/24/2009 | 51.4 | 10/20/2009 | 499.5 | 11/05/2009 | 187.3 |
| 10/21/2004 | 260.3 | 08/26/2001 | 411.6 | 10/11/2009 | 51.1 | 12/07/2009 | 497.8 | 12/07/2009 | 187.1 |
| 10/24/2005 | 258.8 | 06/10/2002 | 410.1 | 04/21/2010 | 50.9 | 01/24/2010 | 498.7 | 02/25/2010 | 186.9 |
| 10/11/2006 | 258.4 | 09/30/2002 | 412.1 | 08/27/2010 | 53.9 | 02/12/2011 | 499.8 | 02/12/2011 | 186.7 |
| 10/30/2007 | 257.5 | 05/28/2003 | 411.5 | 10/14/2010 | 54.6 |  |  |  |  |
| 11/01/2008 | 256.7 | 10/03/2003 | 411.6 |  |  |  |  |  |  |
| 08/24/2009 | 254.5 | 10/21/2004 | 409.5 |  |  |  |  |  |  |
| 10/14/2010 | 256.1 | 10/24/2005 | 409.5 |  |  |  |  |  |  |
|  |  | 10/11/2006 | 410.2 |  |  |  |  |  |  |
|  |  | 10/30/2007 | 409.3 |  |  |  |  |  |  |
|  |  | 11/01/2008 | 409.9 |  |  |  |  |  |  |
|  |  | 06/05/2009 | 408.7 |  |  |  |  |  |  |
|  |  | 08/24/2009 | 408.1 |  |  |  |  |  |  |
|  |  | 09/25/2009 | 407.8 |  |  |  |  |  |  |
|  |  | 08/27/2010 | 410.9 |  |  |  |  |  |  |
|  |  | 10/14/2010 | 411.2 |  |  |  |  |  |  |
