# Supplementary material for: Patterns and Potential Drivers of Dramatic Changes in Tibetan Lakes, 1972–2010
Source: PLoS One. 2014 Nov 5;9(11):e111890. doi: 10.1371/journal.pone.0111890 (PMC4221193; doi:10.1371/journal.pone.0111890)
Supplement: Table S5 — Lake-extent changes in the central plateau (Region C) delineated using Landsat images. (DOCX) [file pone.0111890.s016.docx]

**Table S5** Lake-extent changes in the central plateau (Region C) delineated using Landsat images

| Selin Co | | Qixiang Co | | Nam Co | | Zigetang Co | | Peng Co | |
| --- | --- | --- | --- | --- | --- | --- | --- | --- | --- |
| Date | Area (km^2^) | Date | Area (km^2^) | Date | Area (km^2^) | Date | Area (km^2^) | Date | Area (km^2^) |
| 10/02/1972 | 1628.8 | 10/25/1976 | 153.0 | 12/17/1976 | 1909.9 | 07/16/1973 | 191.5 | 09/30/1972 | 136.0 |
| 02/23/1973 | 1648.7 | 11/06/1999 | 148.6 | 01/19/1989 | 1939.7 | 11/11/1976 | 192.6 | 11/11/1976 | 136.1 |
| 06/11/1973 | 1627.8 | 10/07/2000 | 158.6 | 12/13/1992 | 1929.5 | 11/29/1976 | 192.6 | 11/29/1976 | 135.9 |
| 11/13/1976 | 1635.0 | 07/06/2001 | 160.7 | 01/14/1993 | 1937.6 | 11/30/1976 | 192.8 | 05/28/1977 | 135.9 |
| 12/01/1976 | 1643.0 | 09/24/2001 | 161.1 | 10/30/1999 | 1980.8 | 05/28/1977 | 191.5 | 12/13/1992 | 144.3 |
| 12/19/1976 | 1635.8 | 11/11/2001 | 159.2 | 03/06/2000 | 1971.3 | 07/03/1977 | 191.4 | 09/28/1999 | 150.7 |
| 04/21/1977 | 1649.9 | 05/06/2002 | 159.6 | 11/17/2000 | 1970.9 | 01/19/1989 | 200.0 | 10/30/1999 | 150.9 |
| 01/10/1986 | 1725.5 | 08/26/2002 | 161.3 | 06/13/2001 | 1978.3 | 03/03/1993 | 207.6 | 09/14/2000 | 153.2 |
| 06/30/1990 | 1716.6 | 10/29/2002 | 166.1 | 05/15/2002 | 1994.4 | 09/28/1999 | 209.1 | 11/17/2000 | 153.4 |
| 09/19/1999 | 1797.5 | 11/14/2005 | 176.7 | 12/09/2002 | 1996.7 | 10/30/1999 | 209.3 | 12/19/2000 | 153.2 |
| 03/29/2000 | 1816.5 | 09/14/2006 | 177.5 | 01/10/2003 | 1991.0 | 07/28/2000 | 210.5 | 12/06/2001 | 157.0 |
| 06/17/2000 | 1834.2 | 09/17/2007 | 179.1 | 12/12/2003 | 1994.8 | 09/14/2000 | 211.9 | 02/08/2002 | 156.5 |
| 10/27/2000 | 1903.0 | 10/03/2007 | 179.2 | 02/14/2004 | 2001.3 | 10/16/2000 | 211.8 | 05/15/2002 | 156.9 |
| 11/08/2000 | 1894.3 | 06/02/2009 | 178.7 | 03/17/2004 | 1998.7 | 11/17/2000 | 211.7 | 12/09/2002 | 159.9 |
| 02/12/2001 | 1884.6 | 11/25/2009 | 181.0 | 10/11/2004 | 2013.9 | 12/19/2000 | 212.7 | 04/16/2003 | 159.7 |
| 07/06/2001 | 1926.0 | 04/18/2010 | 179.8 | 05/05/2007 | 2002.1 | 02/05/2001 | 211.4 | 11/04/2004 | 171.7 |
| 07/22/2001 | 1926.5 | 05/04/2010 | 179.5 | 01/08/2008 | 2011.4 | 03/09/2001 | 210.2 | 10/09/2006 | 174.8 |
| 09/24/2001 | 1968.5 |  |  | 02/09/2008 | 2008.8 | 06/13/2001 | 210.8 | 05/05/2007 | 173.2 |
| 10/26/2001 | 1969.8 |  |  | 06/16/2008 | 2013.1 | 10/19/2001 | 214.0 | 11/18/2009 | 175.6 |
| 11/11/2001 | 1953.3 |  |  | 10/06/2008 | 2023.8 | 12/06/2001 | 213.8 |  |  |
| 02/15/2002 | 1964.9 |  |  | 10/22/2008 | 2025.5 | 02/08/2002 | 213.7 |  |  |
| 05/06/2002 | 1969.3 |  |  | 11/07/2008 | 2024.0 | 05/15/2002 | 213.2 |  |  |
| 07/09/2002 | 1985.3 |  |  | 08/30/2009 | 2024.9 | 10/22/2002 | 216.0 |  |  |
| 10/29/2002 | 2012.1 |  |  | 11/18/2009 | 2019.9 | 12/09/2002 | 214.4 |  |  |
| 11/30/2002 | 1998.7 |  |  |  |  | 04/16/2003 | 214.3 |  |  |
| 12/16/2002 | 1997.8 |  |  |  |  | 11/04/2004 | 223.4 |  |  |
| 01/17/2003 | 2002.1 |  |  |  |  | 10/09/2006 | 230.4 |  |  |
| 02/02/2003 | 2006.6 |  |  |  |  | 10/25/2006 | 230.9 |  |  |
| 03/06/2003 | 2000.7 |  |  |  |  | 05/05/2007 | 227.9 |  |  |
| 03/22/2003 | 2001.3 |  |  |  |  | 11/02/2009 | 234.3 |  |  |
| 08/31/2004 | 2179.4 |  |  |  |  | 02/06/2010 | 232.8 |  |  |
| 11/14/2005 | 2242.4 |  |  |  |  | 04/11/2010 | 231.7 |  |  |
| 09/30/2006 | 2275.1 |  |  |  |  | 04/27/2010 | 231.6 |  |  |
| 10/03/2007 | 2284.3 |  |  |  |  |  |  |  |  |
| 11/14/2008 | 2307.7 |  |  |  |  |  |  |  |  |
| 06/02/2009 | 2322.2 |  |  |  |  |  |  |  |  |
| 11/25/2009 | 2338.6 |  |  |  |  |  |  |  |  |
| 01/28/2010 | 2328.7 |  |  |  |  |  |  |  |  |
| 02/13/2010 | 2325.1 |  |  |  |  |  |  |  |  |
| 04/18/2010 | 2329.2 |  |  |  |  |  |  |  |  |
