# Supplementary material for: Patterns and Potential Drivers of Dramatic Changes in Tibetan Lakes, 1972–2010
Source: PLoS One. 2014 Nov 5;9(11):e111890. doi: 10.1371/journal.pone.0111890 (PMC4221193; doi:10.1371/journal.pone.0111890)
Supplement: Table S6 — Lake-extent changes in the northwestern plateau (Region D) delineated using Landsat images. (DOCX) [file pone.0111890.s017.docx]

**Table S6** Lake-extent changes in the northwestern plateau (Region D) delineated using Landsat images

| Lumajiangdong Co | | Bangda Co | | Ze Co | | Jieze Chaka | | Longmu Co | |
| --- | --- | --- | --- | --- | --- | --- | --- | --- | --- |
| Date | Area (km^2^) | Date | Area (km^2^) | Date | Area (km^2^) | Date | Area (km^2^) | Date | Area (km^2^) |
| 10/31/1976 | 345.9 | 12/01/1972 | 102.9 | 10/14/1976 | 112.8 | 12/01/1972 | 104.4 | 12/01/1972 | 96.4 |
| 06/22/1977 | 344.6 | 06/23/1977 | 103.7 | 04/18/1998 | 112.5 | 01/30/1977 | 104.1 | 01/30/1977 | 96.8 |
| 07/28/1977 | 348.4 | 09/29/1999 | 104.6 | 05/04/1998 | 112.5 | 02/17/1977 | 103.9 | 02/17/1977 | 96.9 |
| 09/29/1999 | 347.5 | 10/31/1999 | 104.3 | 05/20/1998 | 112.7 | 06/23/1977 | 104.1 | 06/23/1977 | 96.1 |
| 10/31/1999 | 347.7 | 01/03/2000 | 104.8 | 10/27/1998 | 112.5 | 09/29/1999 | 106.3 | 07/11/1977 | 96.3 |
| 05/10/2000 | 345.4 | 06/27/2000 | 106.1 | 07/18/1999 | 113.8 | 10/31/1999 | 106.2 | 09/29/1999 | 99.4 |
| 09/15/2000 | 351.4 | 12/04/2000 | 109.4 | 08/21/2000 | 115.6 | 01/03/2000 | 106.4 | 10/31/1999 | 99.2 |
| 10/17/2000 | 349.1 | 02/06/2001 | 107.7 | 10/08/2000 | 115.7 | 05/10/2000 | 106.2 | 01/03/2000 | 99.8 |
| 12/04/2000 | 351.2 | 06/30/2001 | 111.1 | 07/07/2001 | 115.4 | 09/15/2000 | 107.2 | 06/27/2000 | 99.3 |
| 03/10/2001 | 351.3 | 09/18/2001 | 113.6 | 04/21/2002 | 115.5 | 10/17/2000 | 106.9 | 09/15/2000 | 100.2 |
| 10/20/2001 | 352.1 | 07/03/2002 | 113.5 | 08/27/2002 | 116.0 | 12/04/2000 | 107.1 | 12/04/2000 | 100.1 |
| 03/29/2002 | 353.4 | 09/21/2002 | 116.5 | 03/07/2003 | 115.9 | 02/06/2001 | 106.6 | 02/06/2001 | 100.0 |
| 07/03/2002 | 352.2 | 01/11/2003 | 116.7 | 05/18/2009 | 117.9 | 09/18/2001 | 108.2 | 03/10/2001 | 100.2 |
| 11/24/2002 | 357.3 | 04/01/2003 | 117.2 | 08/06/2009 | 118.0 | 10/20/2001 | 107.7 | 09/18/2001 | 100.7 |
| 04/01/2003 | 356.3 | 04/17/2003 | 117.3 | 09/07/2009 | 118.2 | 01/08/2002 | 108.0 | 10/20/2001 | 100.2 |
| 04/17/2003 | 356.4 | 09/27/2007 | 126.9 | 09/23/2009 | 118.2 | 03/29/2002 | 108.1 | 01/08/2002 | 100.6 |
| 10/02/2009 | 369.5 | 08/15/2009 | 131.3 | 10/25/2009 | 117.9 | 07/03/2002 | 109.0 | 03/29/2002 | 100.6 |
| 11/03/2009 | 369.1 | 10/02/2009 | 131.6 | 10/28/2010 | 118.8 | 09/21/2002 | 109.4 | 07/03/2002 | 100.4 |
| 03/11/2010 | 369.1 | 07/17/2010 | 131.5 | 11/13/2010 | 118.9 | 11/24/2002 | 109.1 | 09/05/2002 | 101.0 |
| 07/17/2010 | 370.5 |  |  |  |  | 01/11/2003 | 109.0 | 09/21/2002 | 101.1 |
|  |  |  |  |  |  | 04/17/2003 | 109.1 | 11/24/2002 | 101.1 |
|  |  |  |  |  |  | 10/02/2009 | 112.0 | 01/11/2003 | 101.1 |
|  |  |  |  |  |  | 11/03/2009 | 112.5 | 04/17/2003 | 100.6 |
|  |  |  |  |  |  | 11/19/2009 | 112.2 | 09/27/2007 | 102.6 |
|  |  |  |  |  |  | 01/22/2010 | 111.9 | 08/15/2009 | 103.3 |
|  |  |  |  |  |  | 04/28/2010 | 111.9 | 10/02/2009 | 103.5 |
|  |  |  |  |  |  | 07/17/2010 | 112.2 | 11/03/2009 | 103.7 |
|  |  |  |  |  |  |  |  | 01/22/2010 | 103.8 |
|  |  |  |  |  |  |  |  | 02/07/2010 | 103.4 |
|  |  |  |  |  |  |  |  | 03/11/2010 | 103.3 |
|  |  |  |  |  |  |  |  | 07/17/2010 | 103.6 |
