# Supplementary material for: Patterns and Potential Drivers of Dramatic Changes in Tibetan Lakes, 1972–2010
Source: PLoS One. 2014 Nov 5;9(11):e111890. doi: 10.1371/journal.pone.0111890 (PMC4221193; doi:10.1371/journal.pone.0111890)
Supplement: Table S7 — Lake-extent changes in the northeastern plateau (Region E) delineated using Landsat images. (DOCX) [file pone.0111890.s018.docx]

**Table S7** Lake-extent changes in the northeastern plateau (Region E) delineated using Landsat images

| Ulanula Lake | | Xijir Ulan Lake | | Kekexili Lake | | LexieWudan Lake | | Kusai Lake | |
| --- | --- | --- | --- | --- | --- | --- | --- | --- | --- |
| Date | Area  (km^2^) | Date | Area  (km^2^) | Date | Area  (km^2^) | Date | Area  (km^2^) | Date | Area  (km^2^) |
| 07/16/1973 | 544.1 | 07/16/1973 | 373.7 | 07/16/1973 | 310.5 | 10/02/1972 | 235.7 | 10/31/1973 | 268.5 |
| 10/25/1976 | 556.1 | 10/25/1976 | 381.5 | 11/12/1976 | 313.5 | 06/28/1973 | 236.0 | 10/24/1976 | 269.9 |
| 12/18/1994 | 486.2 | 01/10/1989 | 332.5 | 05/29/1977 | 312.8 | 01/10/1989 | 220.8 | 02/27/1977 | 270.1 |
| 09/08/1998 | 506.5 | 12/18/1994 | 312.6 | 08/27/1977 | 316.2 | 11/02/1989 | 225.0 | 01/19/1989 | 254.5 |
| 04/14/2000 | 494.7 | 09/08/1998 | 329.7 | 11/02/1989 | 306.7 | 09/29/1994 | 220.9 | 10/24/1994 | 258.3 |
| 10/07/2000 | 520.8 | 04/14/2000 | 324.6 | 09/29/1994 | 301.2 | 11/06/1999 | 221.1 | 10/30/1999 | 256.2 |
| 09/24/2001 | 536.3 | 10/07/2000 | 341.5 | 04/14/2000 | 301.7 | 04/14/2000 | 220.1 | 04/23/2000 | 255.8 |
| 10/26/2001 | 529.9 | 09/24/2001 | 350.3 | 06/17/2000 | 304.8 | 06/17/2000 | 220.5 | 10/16/2000 | 256.9 |
| 08/26/2002 | 548.5 | 10/26/2001 | 349.9 | 10/07/2000 | 304.6 | 10/07/2000 | 225.1 | 01/07/2002 | 256.3 |
| 03/22/2003 | 553.3 | 08/26/2002 | 359.0 | 09/24/2001 | 305.0 | 05/19/2001 | 222.5 | 03/28/2002 | 256.9 |
| 09/14/2006 | 580.4 | 03/22/2003 | 370.4 | 10/13/2002 | 312.0 | 09/24/2001 | 226.0 | 05/15/2002 | 257.1 |
| 09/17/2007 | 585.2 | 09/14/2006 | 397.9 | 10/03/2007 | 320.6 | 06/23/2002 | 221.9 | 11/07/2002 | 260.9 |
| 10/03/2007 | 586.4 | 09/17/2007 | 404.9 | 08/21/2009 | 323.1 | 10/13/2002 | 237.2 | 12/09/2002 | 259.6 |
| 06/18/2009 | 579.4 | 10/03/2007 | 403.9 | 05/04/2010 | 322.4 | 03/22/2003 | 234.3 | 02/11/2003 | 258.2 |
|  |  | 06/18/2009 | 401.9 | 08/08/2010 | 328.4 | 09/14/2006 | 249.0 | 09/07/2006 | 269.9 |
|  |  |  |  |  |  | 07/31/2007 | 247.7 | 10/09/2006 | 271.3 |
|  |  |  |  |  |  | 10/03/2007 | 250.9 | 10/25/2006 | 270.2 |
|  |  |  |  |  |  | 08/21/2009 | 255.3 | 01/29/2007 | 268.2 |
|  |  |  |  |  |  | 05/04/2010 | 253.2 | 07/24/2007 | 270.4 |
|  |  |  |  |  |  | 08/08/2010 | 260.5 | 08/30/2009 | 278.3 |
|  |  |  |  |  |  |  |  | 04/11/2010 | 277.2 |
|  |  |  |  |  |  |  |  | 04/27/2010 | 278.0 |
|  |  |  |  |  |  |  |  | 09/02/2010 | 287.3 |
