# Supplementary material for: Patterns and Potential Drivers of Dramatic Changes in Tibetan Lakes, 1972–2010
Source: PLoS One. 2014 Nov 5;9(11):e111890. doi: 10.1371/journal.pone.0111890 (PMC4221193; doi:10.1371/journal.pone.0111890)
Supplement: Table S8 — Meteorological stations (117) within and around the Tibetan Plateau used in this study. (DOCX) [file pone.0111890.s019.docx]

**Table S8** Meteorological stations (117) within and around the Tibetan Plateau used in this study

| Station  ID | Station  Name | Latitude  (˚) | Longitude  (˚) | Altitude  (m) | Station  ID | Station  Name | Latitude  (˚) | Longitude  (˚) | Altitude  (m) |
| --- | --- | --- | --- | --- | --- | --- | --- | --- | --- |
| 51777 | Ruoqiang | 39.03 | 88.17 | 887.7 | 55591 | Lasha | 29.67 | 91.13 | 3648.9 |
| 51804 | Tashikuergan | 37.77 | 75.23 | 3090.1 | 55593 | Mozhugangka | 29.85 | 91.73 | 3804.3 |
| 51811 | Sheche | 38.43 | 77.27 | 1231.2 | 55597 | Qongjie | 29.03 | 91.68 | 3741.0 |
| 51818 | Pishan | 37.62 | 78.28 | 1375.4 | 55598 | Zedang | 29.25 | 91.77 | 3551.7 |
| 51828 | Hetian | 37.13 | 79.93 | 1375.0 | 55655 | Nielaer | 28.18 | 85.97 | 4285.0 |
| 51839 | Minfeng | 37.07 | 82.72 | 1409.5 | 55664 | Dingri | 28.63 | 87.08 | 4300.0 |
| 51855 | Qiemo | 38.15 | 85.55 | 1247.2 | 55680 | Jiangzi | 28.92 | 89.60 | 4040.0 |
| 51886 | Mangya | 38.25 | 90.85 | 2944.8 | 55681 | Langkazi | 28.97 | 90.40 | 4432.4 |
| 51931 | Yutian | 36.85 | 81.65 | 1422.0 | 55690 | Cona | 27.98 | 91.95 | 4280.3 |
| 52418 | Dunhuang | 40.15 | 94.68 | 1139.0 | 55696 | Longzi | 28.42 | 92.47 | 3860.0 |
| 52424 | Anxi | 40.53 | 95.77 | 1170.9 | 55773 | Pali | 27.73 | 89.08 | 4300.0 |
| 52436 | Yumenzhen | 40.27 | 97.03 | 1526.0 | 56004 | Tuotuohe | 34.22 | 92.43 | 4533.1 |
| 52447 | Jinta | 40.00 | 98.90 | 1270.5 | 56018 | Zaduo | 32.90 | 95.30 | 4066.4 |
| 52533 | Jiuquan | 39.77 | 98.48 | 1477.2 | 56021 | Qumalai | 34.13 | 95.78 | 4175.0 |
| 52546 | Gaotai | 39.37 | 99.83 | 1332.2 | 56029 | Yushu | 33.02 | 97.02 | 3947.0 |
| 52602 | Lenghu | 38.75 | 93.33 | 2770.0 | 56033 | Maduo | 34.92 | 98.22 | 4272.3 |
| 52633 | Tuole | 38.80 | 98.42 | 3367.0 | 56034 | Qingshuihe | 33.80 | 97.13 | 4415.4 |
| 52645 | Yeniugou | 38.42 | 99.58 | 3219.0 | 56038 | Shiqu | 32.98 | 98.10 | 4239.0 |
| 52652 | Zhangye | 38.93 | 100.43 | 1482.7 | 56043 | Guoluo | 34.47 | 100.25 | 3719.0 |
| 52657 | Qilian | 38.18 | 100.25 | 2787.4 | 56046 | Dari | 33.75 | 99.65 | 3967.5 |
| 52661 | Shandan | 38.80 | 101.08 | 1764.6 | 56065 | Henan | 34.73 | 101.60 | 3515.0 |
| 52674 | Yongchang | 38.23 | 101.97 | 1976.9 | 56067 | Jiuzhi | 33.43 | 101.48 | 3628.5 |
| 52679 | Wuwei | 37.92 | 102.67 | 1531.5 | 56074 | Maqu | 34.00 | 102.08 | 3471.4 |
| 52707 | Xiaozhaohuo | 36.80 | 93.68 | 2767.0 | 56079 | Ruoergai | 33.58 | 102.97 | 3439.6 |
| 52713 | Dachaidan | 37.85 | 95.37 | 3173.2 | 56080 | Hezuo | 35.00 | 102.90 | 2910.0 |
| 52737 | Delingha | 37.37 | 97.37 | 2981.5 | 56093 | Minxian | 34.43 | 104.02 | 2315.0 |
| 52754 | Gangcha | 37.33 | 100.13 | 3311.0 | 56106 | Suoxian | 31.88 | 93.78 | 4022.8 |
| 52765 | Menyuan | 37.38 | 101.62 | 2882.0 | 56109 | Biru | 31.48 | 93.78 | 4192.0 |
| 52787 | Wuqiaoling | 37.20 | 102.87 | 3045.1 | 56116 | Dingqing | 31.42 | 95.60 | 3873.1 |
| 52797 | Jingtai | 37.18 | 104.05 | 1630.9 | 56125 | nangqian | 32.20 | 96.48 | 3643.7 |
| 52818 | Geermu | 36.42 | 94.90 | 2807.6 | 56128 | Leiwuqi | 31.22 | 96.60 | 3810.0 |
| 52825 | Nuomuhong | 36.43 | 96.42 | 2790.4 | 56137 | Changdu | 31.15 | 97.17 | 3306.0 |
| 52833 | Wulan | 36.92 | 98.48 | 2962.0 | 56144 | Delin | 31.80 | 98.58 | 3640.0 |
| 52836 | Dulan | 36.30 | 98.10 | 3191.1 | 56146 | Ganzi | 31.62 | 100.00 | 3377.0 |
| 52856 | Qiabuqia | 36.27 | 100.62 | 2835.0 | 56151 | Banma | 32.93 | 100.75 | 3611.0 |
| 52866 | Xining | 36.72 | 101.75 | 2295.2 | 56152 | Seda | 32.28 | 100.33 | 3922.0 |
| 52868 | Guizhou | 36.03 | 101.43 | 2237.1 | 56167 | Daofu | 30.98 | 101.12 | 2957.2 |
| 52876 | Minhe | 36.32 | 102.85 | 1813.9 | 56172 | Maerkang | 31.90 | 102.23 | 2664.4 |
| 52884 | Gaolan | 36.35 | 103.93 | 1668.5 | 56173 | Hongyuan | 32.80 | 102.55 | 3491.6 |
| 52908 | Wudaoliang | 35.22 | 93.08 | 4612.2 | 56178 | Xiaojin | 31.00 | 102.35 | 2369.2 |
| 52943 | Xinghai | 35.58 | 99.98 | 3323.2 | 56182 | Songpan | 32.65 | 103.57 | 3317.0 |
| 52955 | Guinan | 35.58 | 100.75 | 3108.0 | 56202 | Jiali | 30.67 | 93.28 | 5323.0 |
| 52974 | Tongren | 35.52 | 102.02 | 2491.4 | 56223 | Luolong | 30.75 | 95.83 | 3640.0 |
| 52983 | Yuzhong | 35.87 | 104.15 | 1874.4 | 56227 | Bomi | 29.87 | 95.77 | 2736.0 |
| 52984 | Linxia | 35.58 | 103.18 | 1917.2 | 56228 | Basui | 30.05 | 96.92 | 3260.0 |
| 52986 | Lintao | 35.35 | 103.85 | 1893.8 | 56247 | Batang | 30.00 | 99.10 | 2589.2 |
| 55228 | Shiquanhe | 32.50 | 80.08 | 4278.6 | 56251 | Xinlong | 30.93 | 100.32 | 3080.0 |
| 55248 | Gaize | 32.15 | 84.42 | 4756.0 | 56257 | Litang | 30.00 | 100.27 | 3948.9 |
| 55279 | Bange | 31.38 | 90.02 | 4700.0 | 56307 | Jiacha | 29.15 | 92.58 | 3220.0 |
| 55294 | Anduo | 32.35 | 91.10 | 4800.0 | 56312 | Linzi | 29.67 | 94.33 | 2991.8 |
| 55299 | Naqu | 31.48 | 92.07 | 4507.0 | 56317 | Milin | 29.22 | 94.22 | 2950.0 |
| 55437 | Pulan | 30.28 | 81.25 | 4900.0 | 56331 | Zuogang | 29.67 | 97.83 | 4257.0 |
| 55472 | Shenzha | 30.95 | 88.63 | 4672.0 | 56342 | Mangkang | 29.68 | 98.60 | 3870.0 |
| 55493 | Dangxiong | 30.48 | 91.10 | 4200.0 | 56357 | Daocheng | 29.05 | 100.30 | 3763.0 |
| 55569 | Lazi | 29.08 | 87.60 | 4412.0 | 56434 | Chayu | 28.65 | 97.47 | 2955.0 |
| 55572 | Nanmulin | 29.68 | 89.10 | 4000.0 | 56444 | Deqin | 28.48 | 98.92 | 3319.0 |
| 55578 | Rikaze | 29.25 | 88.88 | 3836.0 | 56533 | Gongshan | 27.75 | 98.67 | 1583.3 |
| 55585 | Nimu | 29.43 | 90.17 | 3809.4 | 56543 | Zhangdian | 27.83 | 99.70 | 3276.7 |
| 55589 | Gongga | 29.30 | 90.98 | 3555.3 |  |  |  |  |  |
